# Tuning the morphology and energy levels in organic solar cells with metal–organic framework nanosheets

**DOI:** 10.1038/s41598-024-80007-y

**Published:** 2024-11-28

**Authors:** Kezia Sasitharan, Johannes Frisch, Jaroslav Kuliček, Ahmed Iraqi, David G. Lidzey, Marcus Bär, Bohuslav Rezek, Jonathan A. Foster

**Affiliations:** 1 Department of Chemistry, The University of Sheffield, Dainton Building, Brook Hill, Sheffield, S3 7HF UK; 2 Centre for Advanced Photovoltaics, Faculty of Electrical Engineering, Czech Technical University in Prague, 16000 Prague, Czech Republic; 3 Interface Design, Helmholtz-Zentrum Berlin für Materialien und Energie GmbH (HZB), 12489 Berlin, Germany; 4 Energy Materials In‐Situ Laboratory Berlin (EMIL), HZB, 12489 Berlin, Germany; 5 Department of Physics and Astronomy, The University of Sheffield, Hicks Building, Hounsfield Road, Sheffield, S3 7RH UK; 6 Department of Chemistry and Pharmacy, Friedrich-Alexander-Universität Erlangen-Nürnberg (FAU), 91054 Erlangen, Germany; 7 Department X-ray Spectroscopy at Interfaces of Thin Films, Helmholtz Institute Erlangen-Nürnberg for Renewable Energy (HI ERN), 12489 Berlin, Germany

**Keywords:** Materials for energy and catalysis, Metal-organic frameworks

## Abstract

Metal–organic framework nanosheets (MONs) have proved themselves to be useful additives for enhancing the performance of a variety of thin film solar cell devices. However, to date only isolated examples have been reported. In this work we take advantage of the modular structure of MONs in order to resolve the effect of their different structural and optoelectronic features on the performance of organic photovoltaic (OPV) devices. Three different MONs were synthesized using different combinations of two porphyrin-based ligands meso-tetracarboxyphenyl porphyrin (TCPP) or tetrapyridyl-porphyrin (TPyP) with either zinc and/or copper ions and the effect of their addition to polythiophene-fullerene (P3HT-PC_71_BM) OPV devices was investigated. The power conversion efficiency (PCE) of devices was found to approximately double with the addition of MONs of Zn_2_(ZnTCPP) -4.7% PCE, 10.45 mA/cm^2^ short-circuit current density (*J*_SC_), 0.69 open-circuit voltage (*V*_OC_), 64.20% fill-factor (FF), but was unchanged with the addition of Cu_2_(ZnTPyP) (2.6% PCE, 3.68 mA/cm^2^ *J*_SC_, 0.59 *V*_OC_, 46.27% FF) and halved upon the addition of Cu_2_(CuTCPP) (1.24% PCE, 6.72 mA/cm^2^ *J*_SC_, 0.59 *V*_OC_, 56.24% FF) compared to devices without nanosheets (2.6% PCE, 6.61 mA/cm^2^ *J*_SC_, 0.58 *V*_OC_, 56.64% FF). Our analysis indicates that there are three different mechanisms by which MONs can influence the photoactive layer – light absorption, energy level alignment, and morphological changes. Analysis of external quantum efficiency, UV–vis and photoelectron spectroscopy data found that MONs have similar effects on light absorption and energy level alignment. However, atomic force and Raman microscopy studies revealed that the nanosheet thickness and lateral size are crucial parameters in enabling the MONs to act as beneficial additives resulting in an improvement of the OPV device performance. We anticipate this study will aid in the design of MONs and other 2D materials for future use in other light harvesting and emitting devices.

## Introduction

Organic photovoltaics (OPV) have huge potential as a sustainable technology due to their ease of processability, high absorption co-efficient and flexibility^1–5^. Termed “bulk heterojunction,” the active layer of these devices consists of a blend of two organic materials with different optoelectronic properties for providing efficient light absorption, a suitable donor–acceptor blend to facilitate exciton dissociation, and charge transport pathways for energy harvesting^6–10^. Currently, the power-conversion efficiencies of OPVs has reached 20% under 1 sun illumination, thanks to significant progress in development of donor polymers and non-fullerene acceptors, and their morphology control^11–13^. Organic semiconductors typically show low charge carrier mobility and therefore the bulk heterojunction blend of donor–acceptor components is usually thin to achieve better charge collection^14–16^. One strategy to enable complementary absorption and enhanced photovoltaic conversion is to create tandem structures with multiple absorption layers^17^. However, this approach is accompanied with increased fabrication costs and complexity^18^. Ternary organic solar cells with an auxiliary third component incorporated into the donor/acceptor photoactive system is a promising approach that has been investigated extensively to broaden the absorption range of OPVs^19–22^.

A typical ternary blend consists of an additional donor or acceptor component, generally expected to have complementary absorption. Besides the enhanced light absorption, the additives can affect other factors, such as morphology and charge carrier mobility, that can greatly improve the power conversion efficiency (PCE) values^23^. Various third components investigated in the ternary bulk heterojunctions include donor polymers, small molecules, dye molecules, fullerene derivatives as a second acceptor, quantum dots and various semiconducting nanomaterials^24–26^. A key requirement of these ternary components is a proper energy level alignment to ensure loss-less charge transport pathways avoiding the formation of charge carrier recombination centers^27–29^.

Metal–organic framework (MOF) nanosheets (MONs) are an emerging class of two-dimensional materials with a modular structure in which organic linkers are coordinated to metal ions^30,31,64^. These materials have shown enormous potential in a wide range of applications including sensing, catalysis, separation membranes, energy harvesting, and storage^32–35^. In recent years, these materials have been investigated in organic solar cells and related electronic devices with remarkable results, thanks to their nanoscopic dimensions and tunable optoelectronic properties^36^. For example, a twofold enhancement in device lifetime was observed when bis(dithiolato)nickel MONs were incorporated as hole buffer layer in an OLED^37^. The first example of using MONs within a functional solar cell was reported in 2018 and involved using a tellurophene based MON within the electron extraction layer^38^. Other reports have used porphyrin based MONs mixed with fullerenes to create liquid-junction solar cells^39^ and demonstrated a dopant free hole transport layer for perovskite solar cells^40^ or an electron extraction layer employed at the perovskite/cathode interface reducing the leakage of toxic lead ions^41^.

We recently demonstrated the first use of MONs within the photoactive layer of OPVs, where the incorporation of Zn_2_(ZnTCPP) MONs (where TCPP = *meso-tetracarboxyphenyl porphyrin)* into a P3HT-PC_61_BM solar cell increased the PCE by a factor of almost two^42,43^. A detailed analysis of this system showed that the MONs acted as templates, which increased the crystallinity of the donor polymer and thereby resulted in a more balanced charge mobility and improved device performance metrics. We followed up on these results by evaluating Zn_2_(ZnTCPP) MONs as additives in a range of OPV systems and found a significant improvement in other semi-crystalline donor polymers as well, one of it being PffBT4T2OD where we achieved a remarkable 12.3% power conversion efficiency – the highest reported fullerene-based OPV device to date^44^. Through the detailed characterization undertaken as part of these studies we were able to build up a good understanding of the effect of the nanosheets on the surrounding material and so predict which devices would benefit from nanosheet addition. However, we were able to say very little about which structural, optoelectronic, and/or nanoscopic features of the nanosheets were responsible for this record PCE and what could be done to further enhance these effects.

In this work we explore the effect of three types of porphyrin-based MONs (Cu_2_(CuTCPP), Cu_2_(ZnTPyP) where TPyP = tetrapyridyl porphyrin, and Zn_2_(ZnTCPP)) in order to understand the effect of different combinations of metal ions and ligands on device performance. We investigate OPV performance on addition of the MONs, and perform energy level alignment studies. The power conversion efficiency (PCE) of devices was found to approximately double with the addition of MONs of Zn_2_(ZnTCPP) (4.7% PCE, 10.45 mA/cm^2^ *J*_SC_, 0.69 *V*_OC_, 64.20% FF), but was unchanged with the addition of Cu_2_(ZnTPyP) (2.6% PCE, 3.68 mA/cm^2^ *J*_SC_, 0.59 *V*_OC_, 46.27% FF) and halved upon the addition of Cu_2_(CuTCPP) (1.24% PCE, 6.72 mA/cm^2^ *J*_SC_ , 0.59 *V*_OC_, 56.24% FF) compared to devices without nanosheets (2.6% PCE, 6.61 mA/cm^2^ *J*_SC_, 0.58 *V*_OC_, 56.64% FF). We observe that Zn_2_(ZnTCPP) simultaneously improves all device performance parameters (short circuit current density, J_*SC*_; open circuit voltage, V_*OC*_; fill factor, FF; and PCE). Using photoelectron spectroscopy, we study whether the energy level alignment of the different MONs within the ternary blend changes. In addition, we compare the morphology of the different bulk heterojunction systems using atomic force and Raman microscopy to further understand the origin of the improved energy conversion in the Zn_2_(ZnTCPP) based ternary system. This work demonstrates that along with the ideal positioning of the MON frontier energy levels with respect to the donor and the acceptor, it is necessary to achieve a favorable active layer morphology to generate a significant improvement in device performance.

## Results

### Synthesis and characterization of MONs

**Fig. 1:**
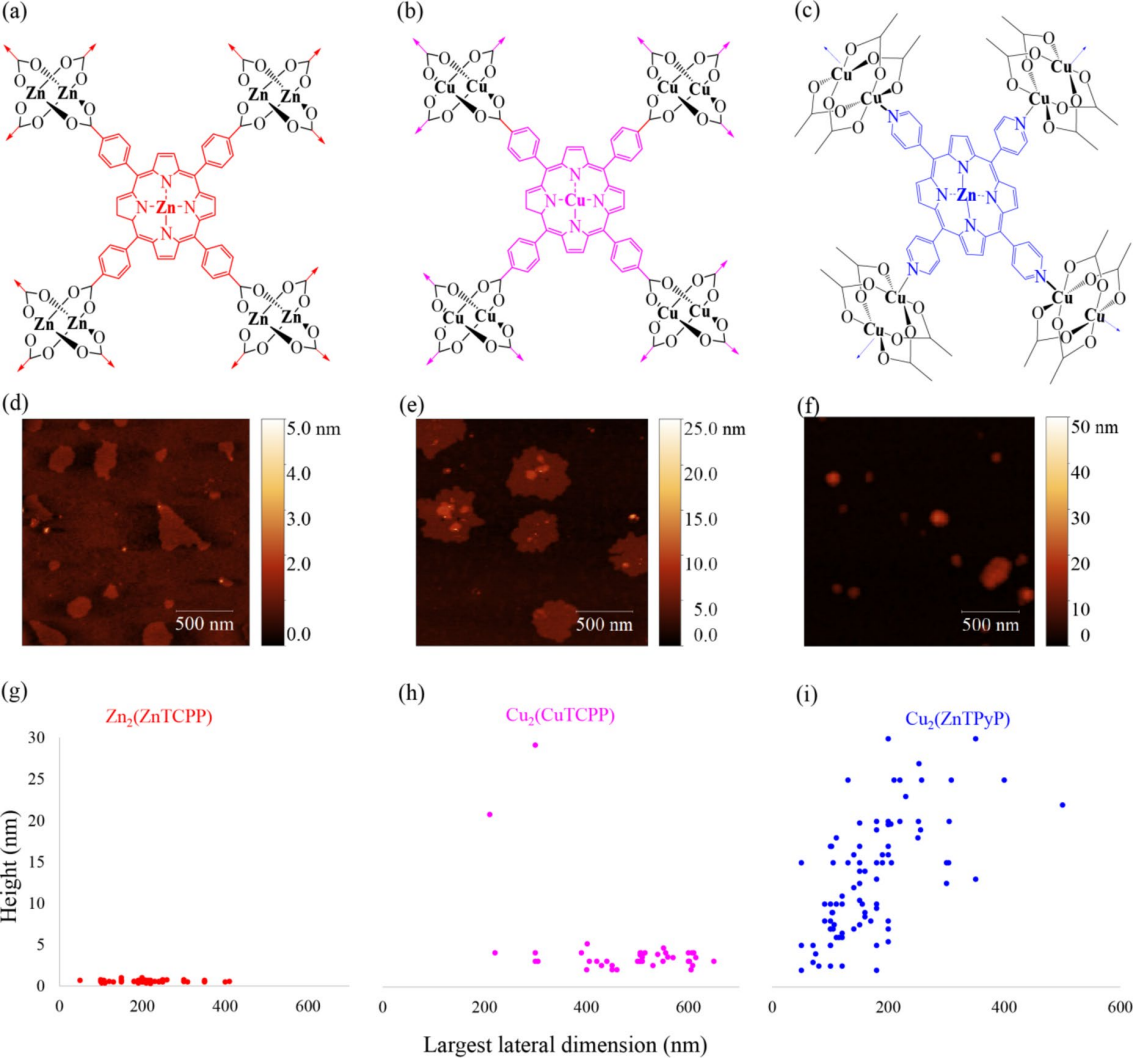
Structure (**a**–**c**) and AFM images (**d**–**f**) of Zn_2_ZnTCPP, Cu_2_(CuTCPP) and Cu_2_(ZnTPyP) MONs used in this work, (**g**–**i**) scatter plots of the height of the MONs (in nm) vs the largest lateral dimension in nm.

Three different porphyrin based MONs were selected for investigation containing different combinations of ligands and metal ions, as shown in Fig. 1a–c. We and others have previously reported the synthesis of Zn_2_(ZnTCPP) which was one of the earliest MONs to be reported and has been widely used for applications ranging from photosensitization to separation^45^. MONs of Cu_2_(CuTCPP) were first reported by Kitagawa and co-workers and since we began our studies has been widely investigated for catalysis, CO_2_ reduction, and sensing applications^46–49^. We are only aware of one previous report of MONs of Cu_2_(ZnTPyP) although it has more extensively been studied for 2D molecular assemblies created by Langmuir–Blodgett technique^50^. It is worth noting that the majority of syntheses of porphyrin based MONs report a “bottom-up” approach in which polyvinylpyridine (PVP) is added during MOF growth to inhibit growth in a third dimension in order to produce nanosheets. Anticipating that PVP could interfere with device performance, we previously developed a surfactant free “top-down” approach in which ultrasound is used to exfoliate the layered Zn_2_(ZnTCPP) MOF resulting in monolayer nanosheets. Here we adapt the same approach to create analogous nanosheets of the other two systems.

In a typical reaction, TCPP was heated with the metal precursor in a DMF: ethanol mixture at 80℃ for 24 h, resulting in a black microcrystalline MOF as the product.Zinc nitrate and copper nitrate were used as the metal precursors for Zn_2_(ZnTCPP) and Cu_2_(CuTCPP), respectively. For the third system, Cu_2_(ZnTPyP), we adapted a method reported previously for a 2D Cu MOF formed by mixing TPyP with copper acetate in chloroform^51^. As these are heterometallic systems, the TPyP ligand was first metallated at its centre by reacting it with zinc nitrate in a 1:1 stoichiometric ratio. The resulting ZnTPyP ligand was then heated with copper acetate to form Cu_2_(ZnTPyP) MOF where all the four pyridyl groups of ZnTPyP are coordinated to four different Cu(II) atoms of the Cu_2_(OAc) units.

In all cases, powder X-ray diffraction patterns of the as-synthesized MOFs showed good agreement with the previously reported single-crystal structures as detailed in the experimental section in the Electron Supplementary Information (ESI Fig. S1–S3). Zn_2_(ZnTCPP) and Cu_2_(CuTCPP) have isoreticular layered structures in which four TCPP molecules are connected via their carboxylic acid moieties to two metal ions to create a metal-paddle wheel motif (PW) with solvent molecules (water or DMF) coordinated to the axial positions of the PW. The layered sheets are stacked in an AB pattern with the metal atoms in the centre of the porphyrin rings being aligned with the metal atoms in the paddlewheel nodes forming a 2D MOF structure. In contrast, Cu_2_(ZnTPyP) has an inverted paddlewheel morphology in which the pyridyl ligands connect in two-dimensions via the axial positions of copper paddle wheels with the equatorial positions capped by acetate ligands. The nanosheets are hetero-metallic with the central porphyrin site occupied by zinc ions and layers stack in an AB sequence such that the zinc atoms in the middle of the TPyP rings are aligned with the copper atoms in the paddlewheel nodes (see Fig. 1a–c).

The three layered MOFs were suspended in ethanol (5 mg/6 ml) and sonicated for 1 h in a temperature controlled ultrasonic bath followed by centrifugation at 1500 rpm for 10 min to remove the larger unexfoliated particles. UV–Vis spectra of the exfoliated MONs in ethanolic suspensions (ESI Figs. S4–S6) show them as strongly absorbing with a characteristic π-π* soret band (423 nm for Zn_2_(ZnTCPP), 431 nm for Cu_2_(CuTCPP), 422 nm for Cu_2_(ZnTPyP) and Q bands. The extinction coefficients of the exfoliated MONs were 29,278 dm^3^ mol^–1^ cm^–1^ for Zn_2_(ZnTCPP), 2134 dm^3^ mol^–1^ cm^–1^ for Cu_2_(CuTCPP)-and 17,778 dm^3^ mol^–1^ cm^–1^ for Cu_2_(ZnTPyP). The supernatant consisting of each of the exfoliated MONs was collected and 20 μL of it was deposited onto freshly cleaved mica (preheated to 80℃) for atomic force microscopy (AFM) imaging as shown in Fig. 1d–f. Figure 1g–i show scatter plots of height (in nm) vs largest lateral dimension (in nm) of the different MONs using data taken from Fig. 1d–f and ESI, Figs. S7–S21. AFM particle size analysis showed that the Zn_2_(ZnTCPP) MONs were consistent in size with our previous paper and are 0.5–1 nm thin (RMS = 0.63 nm) with a lateral size distribution ranging between 50 and 400 nm (RMS = 224.42 nm). Based on the expected inter-layer spacing from the known crystal structures, the Zn2(ZnTCPP) MONs are predominantly monolayer thick. The Cu_2_(CuTCPP) MONs were largely found to be between 4 and 5 nm in height (~ 3 layers thick) (RMS = 6.29 nm) and between 400 and 600 nm (RMS = 495.36 nm) in lateral dimension. The Cu_2_(ZnTPyP) MONs were found to be considerably thicker, between 15 and 40 nm in height (RMS = 23.23 nm) and their lateral dimensions were found to be between 50 and 400 nm (RMS = 216.93 nm). Under these conditions, Zn_2_(ZnTCPP) nanosheets formed the highest aspect ratio nanosheets, with Cu_2_(CuTCPP) being few layer nanosheets with higher lateral dimensions and Cu_2_(ZnTPyP) being considerably thicker with smaller lateral dimensions. These differences could be due to differences in the strength of in-layer or inter-layer interactions formed with different metal-ions/ligands. It is worth noting that the same conditions were used for each system, but these had previously been optimized for Zn_2_(ZnTCPP) so further optimization of the other two nanosheets could well also yield monolayer nanosheets. However as these nanosheets are comparable to other MONs reported by us and others we proceeded with these systems without further optimization.

### Incorporation into ternary OPV devices

**Fig. 2:**
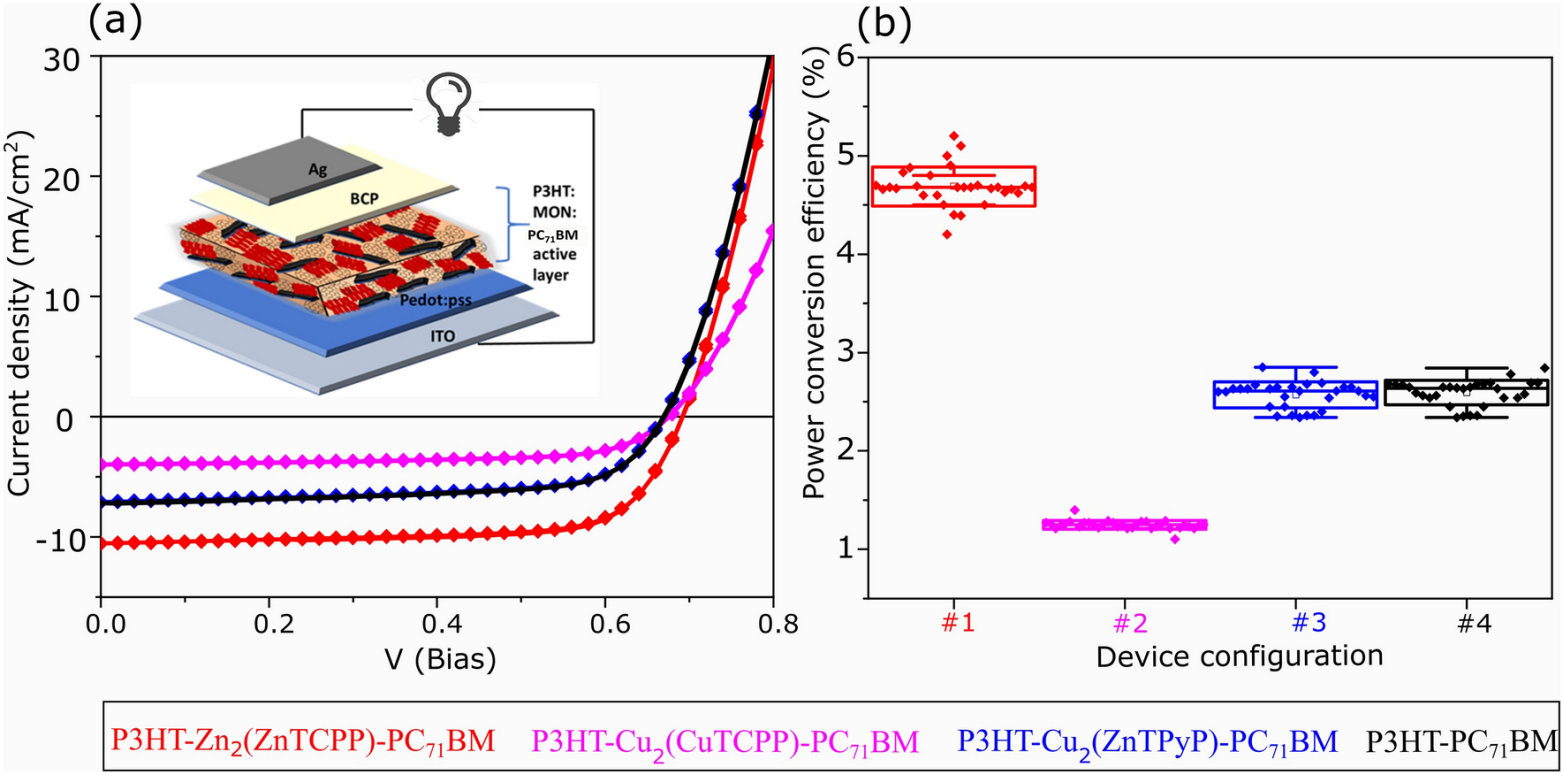
(**a**) Current–voltage curves and (**b**) PCE averaged over 30 devices for each P3HT-MON-PC_71_BM combination are plotted as a function of the active layer composition, please consider the color code presented in the legend below the panels.

The effect of the different MONs as ternary additives within bulk heterojunction OPV devices was investigated in optimised P3HT-PC_71_BM host devices. For the active layer, MONs were mixed with P3HT-PC_71_BM with a loading of 20% by weight (methods detailed in supplementary information) and the resulting ternary blend was spin coated onto PEDOT:PSS coated ITO, followed by BCP/Ag as the back contact (ESI, Fig. S22). Table 1 details the PV performance metrics. Representative current–voltage responses (J-V curve) of the devices are shown in Fig. 2a and the power conversion efficiency (PCE, %) averaged over 30 devices for each P3HT-MON-PC_71_BM combination are plotted as a function of the active layer composition in Fig. 2b. The analysis of the other performance parameters are plotted in ESI, Fig. S23 and PV parameters of bilayer devices are shown in ESI Figs. S24–S29 and Table S1–S7. The improvement observed for the Zn_2_(ZnTCPP) device confirms our previous result^42^. However, the addition of Cu_2_(CuTCPP) was found to substantially decrease device performance whilst.

**Table 1:** The average PV parameters and their standard deviation values calculated from 30 devices for each P3HT-MON-PC_71_BM configuration used in this study.

Device configuration	− J_*SC*_ (mAcm^2^)	V_*OC*_ (V)	FF (%)	PCE (%)
P3HT-Zn_2_(ZnTCPP)-PC_71_BM	10.45 ± 0.21	0.69 ± 0.01	64.20 ± 0.90	4.69 ± 0.20
P3HT-Cu_2_(CuTCPP)-PC_71_BM	3.68 ± 0.2	0.59 ± 0.01	46.27 ± 1.14	1.24 ± 0.04
P3HT-Cu_2_(ZnTPyP)-PC_71_BM	6.72 ± 0.25	0.59 ± 0.01	56.24 ± 1.10	2.57 ± 0.13
P3HT-PC_71_BM	6.61 ± 0.33	0.58 ± 0.01	56.64 ± 1.06	2.59 ± 0.12

**Fig. 3:**
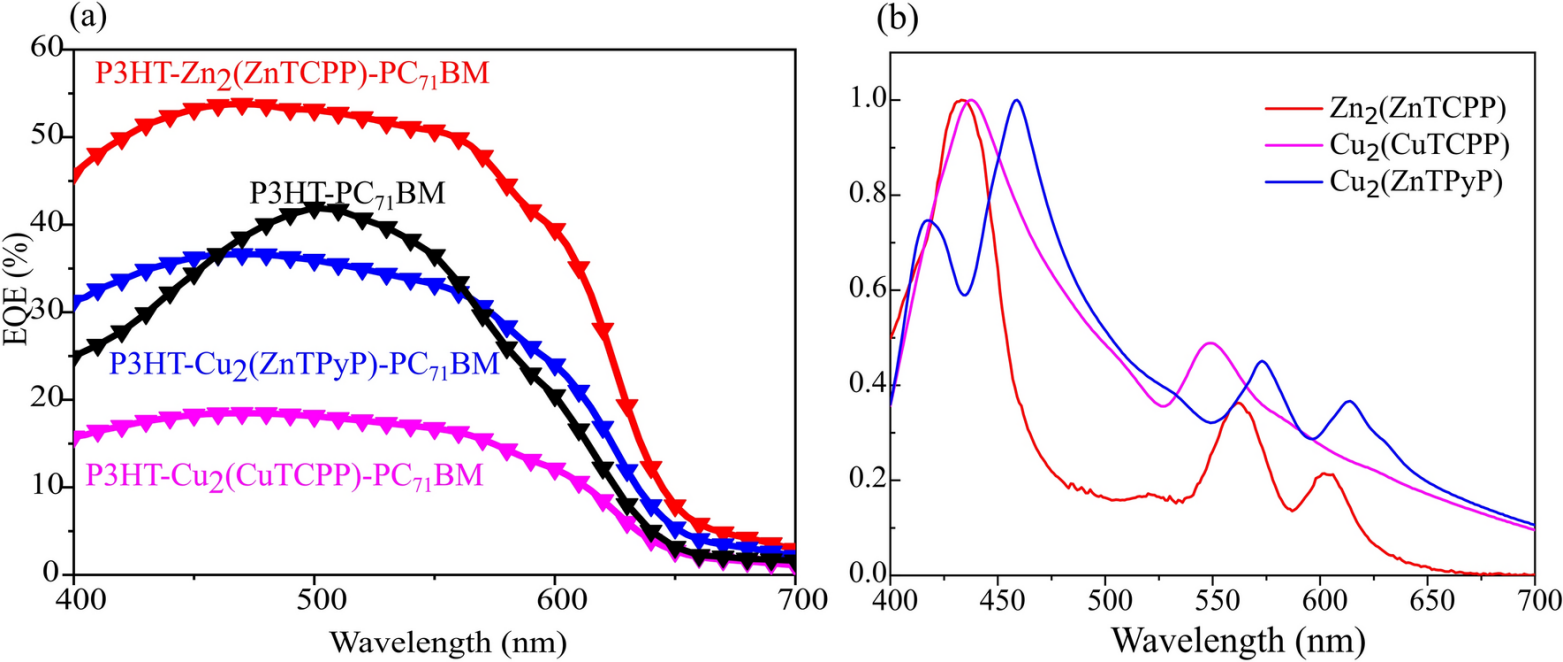
(**a**) External quantum efficiency (EQE) of the P3HT-additive-PC_71_BM devices without and with different ternary additives, and (**b**) thin-film absorption spectra of the pure MONs cast from ethanol.

Cu_2_(ZnTPyP) resulted in no change in device efficiency compared to the P3HT-PC_71_BM device without ternary additive. Zn_2_(ZnTCPP) causes an increase in absorption in the films as compared to P3HT-PC_71_BM blend which contributes to an increase in J_*SC*_ by 4 mA/cm^2^. Furthermore, an observed increase in V_*OC*_ by 0.3 V and FF by ~ 8% suggests a reduced recombination rate within the ternary bulk heterojunction, cumulatively leading to an improved PCE of 4.69%. Both Cu_2_(CuTCPP) and Cu_2_(ZnTPyP) have an unchanged V_*OC*_ compared to the pristine P3HT-PC_71_BM devices. This indicates that the effective donor–acceptor energy gap (i.e., the energy difference between the onset of the Fullerene LUMO and the P3HT HOMO) presumably remained unaffected by the presence of the MONs. While Cu_2_(ZnTPyP) did not cause any statistically significant reduction in device performance, Cu_2_(CuTCPP) reduced the J_*SC*_ by 3 mA/cm^2^ and the FF by ~ 10%. This large decrease in FF along with the decreased quantum efficiency (see discussion in conjunction to Fig. 3, below) for this device suggests possible differences in the charge transport pathways and (or) a modification of the photoactive nanostructure with the incorporation of different types of MONs.

### Quantum efficiency and absorption

External quantum efficiency measurements (EQE) shown in Fig. 3a were conducted to gain insight into the photoresponse of the ternary systems across the wavelength range 400 nm to 700 nm. The Jsc calculated from EQE are consistent with the measured values with < 5% error. When compared with the P3HT-PC_71_BM devices, all the P3HT-MON-PC_71_BM systems demonstrate a broader and increased EQE spectrum compared to the reference devices without nanosheets which is attributed to additional absorption from the porphyrin units (see Fig. 3b). In line with our previous results, the P3HT-Zn_2_(ZnTCPP)-PC_71_BM devices show a strong and uniform photo-response between 400 and 600 nm, with a maximum EQE at 55%^42^. Significantly, there is an enhanced photoresponse in the 400 to 450 nm region which corresponds to the S-absorption band of porphyrins and additional photoresponse in the 600 to 650 nm region, attributed to the increased crystallinity of P3HT which we describe in our previous work^42^.

The P3HT-Cu_2_(ZnTPyP)-PC_71_BM and P3HT-Cu_2_(CuTCPP)-PC_71_BM devices show a similar uniform photoresponse over the 400 to 600 nm spectral region as the P3HT-Zn_2_(ZnTCPP)-PC_71_BM cell, but with much reduced intensity with the maximum EQE being only 36 and 18%, respectively. This is surprising, as the absorption spectrum in Fig. 3b shows absorption normalized to the maximum intensity. For the Cu_2_(ZnTPyP), we observe in addition to the enhanced absorption contribution in the S band region, a pronounced shoulder at 610 nm which corresponds to a second Q-band associated with this MONs and increased scattering due to the larger particle size.

These findings confirm that the MONs are photoactive in the blends, with the porphyrin units contributing to net light absorption. However, this benefit does apparently not result in a higher EQE. In contrast, the photoresponse in the primary absorption region of P3HT-PC_71_BM at 500 nm is weakened by 5% with the incorporation of Cu_2_(ZnTPyP) and by 30% in the case of Cu_2_(CuTCPP). This indicates charge carrier extraction issues, presumably due to changes in the active layer morphology and charge transport pathways within these devices – an observation that will be explored below.

### Photoemission studies

**Fig. 4:**
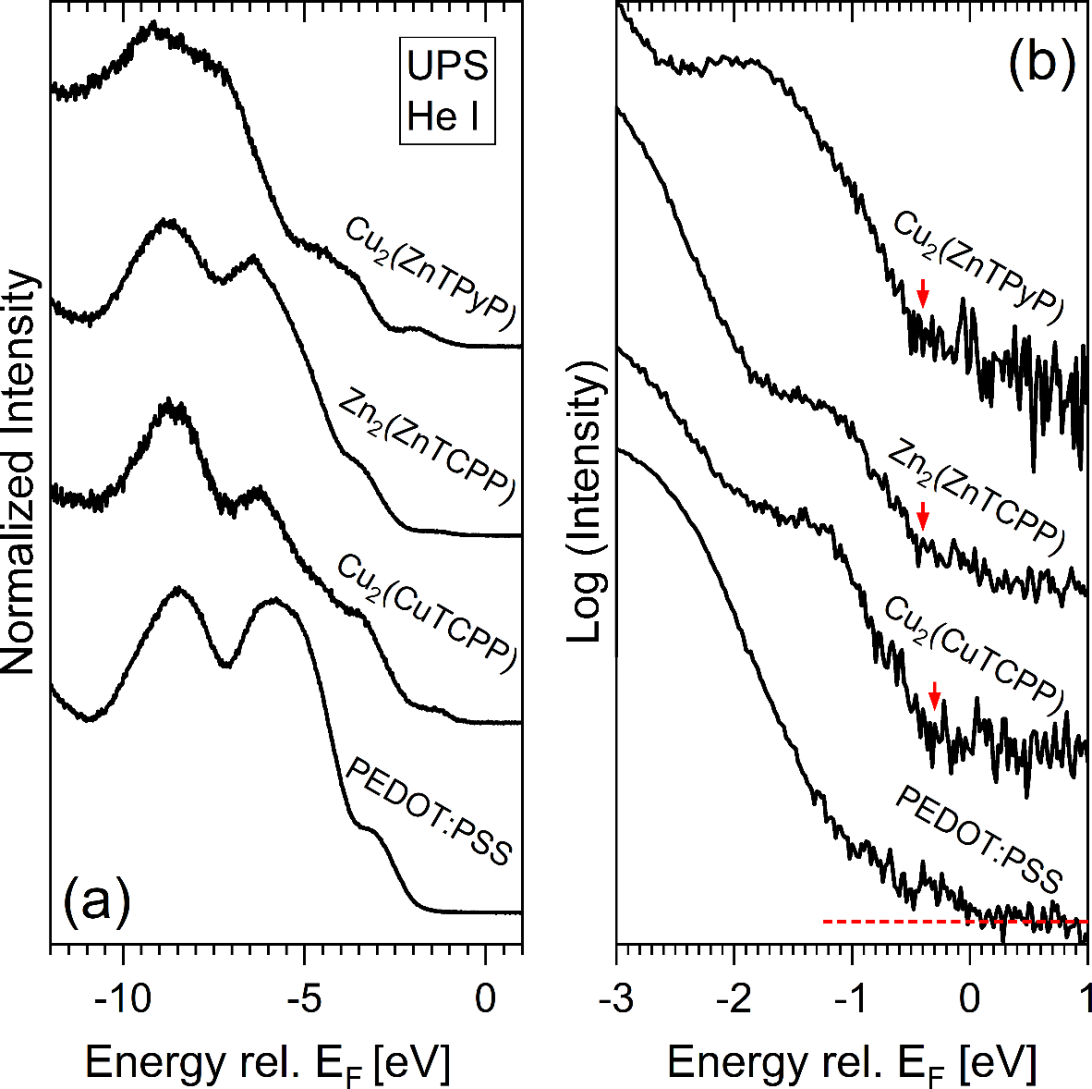
(**a**) UPS (He I) valence band spectra of PEDOT:PSS without (bottom spectrum) and with different spin-coated MON additives. (**b**) Magnified region of the same spectra on logarithmic scale for inspection of the leading edge to derive the valence band maxima (VBM), indicated by the red arrows. For the spectrum of PEDOT:PSS the background (red dashed line) is indicated, revealing that there is occupied density of states straight up to the Fermi level (E_F_) at 0 eV.

UV photoelectron spectroscopy (UPS) experiments were performed in conjunction with X-ray photoelectron spectroscopy (XPS) measurements to gain insight whether the studied MONs differently affect the energy level alignment within the blend. The respective UPS spectra of Cu_2_(ZnTPyP), Zn_2_(ZnTCPP), and Cu_2_(CuTCPP) spin-coated on PEDOT:PSS/ITO substrates are shown in Fig. 4. We use PEDOT:PSS here as the substrate of choice, as it (in contrast to P3HT or PC_71_BM) only has a very low density of occupied states in the region close to the Fermi edge (E_F_), allowing the identification of the MONs related valence band maximum (VBM) values. Note that since MONs are anisotropic materials they have been spin-coated from suspensions rather than solutions (see ESI 2.9 for details) and thus the resulting films do not cover the PEDOT:PSS substrate homogeneously, but MON islands are formed. The incomplete coverage is also corroborated by the XPS survey spectra of the samples (see Fig. S30, ESI). Most prominently, the S 2p signal of the substrate can be observed in all survey spectra. However, the Cu 2p and/or Zn 2p (see Fig. S31, ESI) detail spectra clearly indicate the deposition of the MONs. It should be noted that the morphology of the surface plays a very important role in determining the work function and the resulting ionisation energy and thus the derived values can only be an estimate for the energetic situation in the blend where the MONs morphology is expected to be different.

The UPS spectrum of the uncovered PEDOT:PSS substrate (bottom of Fig. 4a) is in agreement with other measurements^52^ for (thermally) stressed PEDOT:PSS, consistent with the annealing treatment used after spin coating (see ESI). Spectra for the different MONs spin-coated onto PEDOT:PSS showed similar spectral features at first glance, indicating incomplete coverage of the MONs. Close inspection of the energy region close to E_F_ on a semi-logarithmic scale in Fig. 4b, reveals PEDOT:PSS density of occupied states up to EF and clear MONs related VBM features. Using the intersection of the linear extrapolation of the leading spectral edge and of the background signal, we derive VBM values of 0.3, 0.4, and 0.4 (± 0.1) eV (indicated by the red arrows in Fig. 4b) for Cu_2_(CuTCPP), Zn_2_(ZnTCPP) and Cu_2_(ZnTPyP) respectively. Note that the VBM determination on a linear intensity scale gives the same values within the experimental uncertainty.. Thus, the energetic landscape within the ternary blend active layer is presumably very similar for the three different MONs and hence does not explain the different performance of the corresponding solar cell devices (see Fig. 2). A more detailed discussion can be found in the ESI in conjunction with the analysis of the UPS derived MONs’ work function values depicted in Fig. S32a. The optical band gaps are given in Fig. S32b.

### Morphology – AFM and Raman analysis

**Fig. 5:**
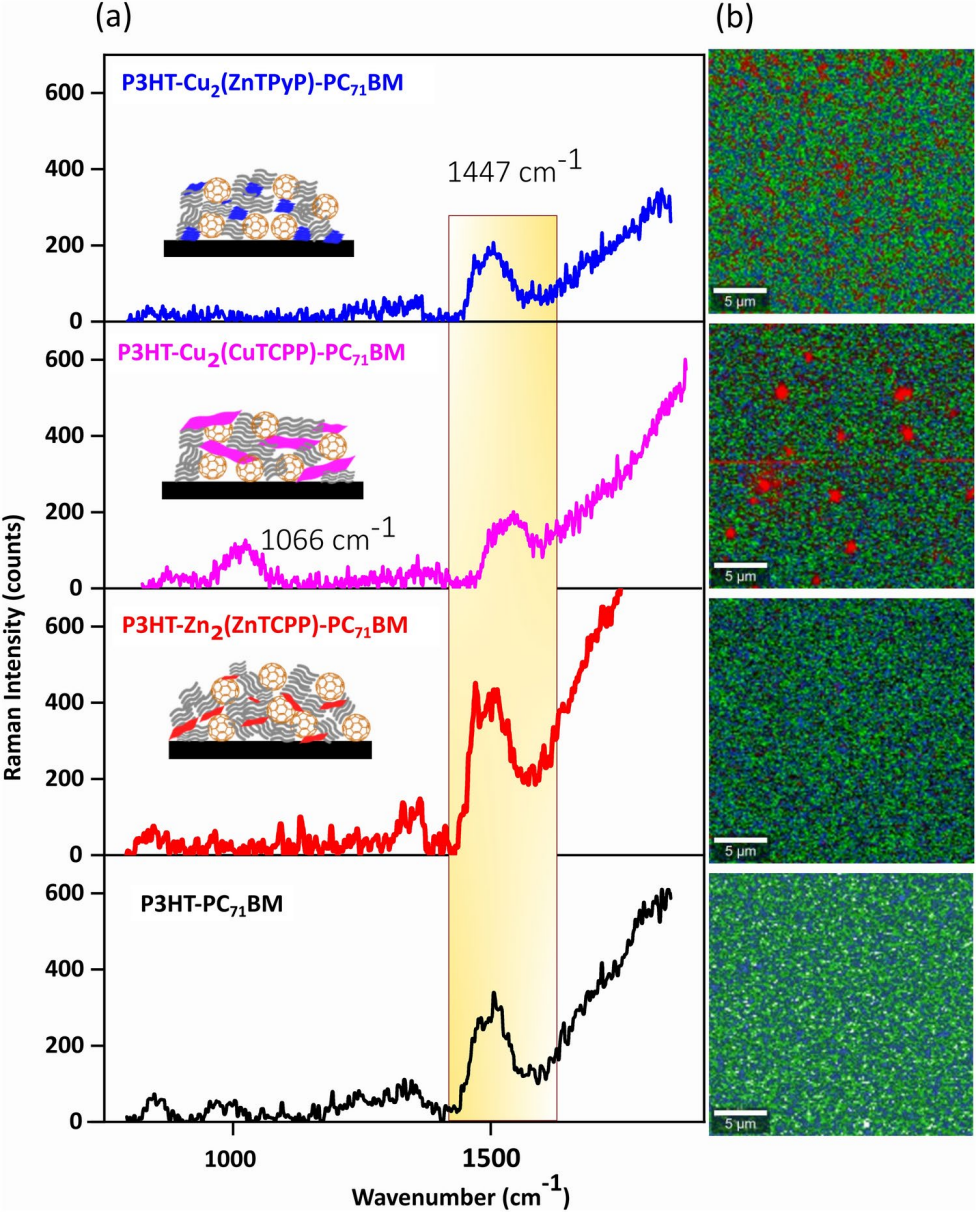
Raman spectra and mapping of the ternary blend active layer. Panel (**a**) shows the acquired single spectra and and panel (**b**) shows the mapped images.

AFM imaging of the ternary blend active layers (ESI, Figs. S33–S36) was conducted to provide more insights into the morphology of the bulk heterojunctions. A spatial Fast-Fourier transform was applied to the images to extract the grain size (phase separation length scales) according to literature methods with the detailed evaluation result listed in ESI, Table S7. As reported previously, the grain size in P3HT-PC_71_BM active layer are approx 10 nm whereas the grain sizes were found to reduce to about 8.7 nm^42^ with the addition of Zn_2_ZnTCPP. For devices containing Cu_2_(CuTCPP) and Cu_2_(ZnTPyP) in the active layer, the grain sizes are larger than that found in the control devices, upto 20 nm for Cu_2_(ZnTPyP) and up to 47.4 nm for the Cu_2_(CuTCPP) containing films. Raman mapping was performed to gain information about the compositions of the ternary blend active layers. The peak at 1447 cm^–1^ (Fig. 5a) is attributed to symmetric C = C stretch and the peak at 1325 cm^–1^ is the C–C intra-ring stretch of P3HT^53–61^. In accordance with literature, the high intensity of P3HT band 1447 cm^–1^ was used to determine the distribution of P3HT in the ternary blend. In the maps shown in ESI, Fig. S37, the yellow domains represent the highest peak intensity and thus the highest relative concentration of P3HT, whereas the black domains represent the lowest P3HT concentration. Because of the high intensity of the P3HT bands (related to the high crystallinity and close packing of P3HT), the much lower intensity of the PC_71_BM or MON bands were difficult to discern except in the case of P3HT-Cu_2_(CuTCPP)-PC_71_BM which shows the Raman peak corresponding to the porphyrin unit at 1027 cm^–1^ v(Cα-Cµ) and 1066 cm^–1^ δ(Cβ-H)^62^. We attribute this to the comparatively larger lateral size of the Cu_2_(CuTCPP) MONs.

In the Raman mapping from the blends, the contribution from MON and PC_71_BM were evaluated using the “true component analysis de-mixing of spectra”- a data processing tool of the Project FIVE 5.1 software (WITec GmbH, Germany)^63^. From the spectral data set, true component analysis finds components by selecting the most intense data set (P3HT in this case) as the base spectrum and calculates the residual image. This is followed by subsequent cycles of adding components and calculating the corresponding residual images and creating intensity distribution images (component mapping), with corresponding average component spectra that supports de-mixing of spectra (to show the distribution of different components). Component maps can be found in ESI Fig. S38–41. This method was applied to obtain the Raman maps shown in Fig. 5b right side, where the MON regions are represented in red, while the P3HT-PC_71_BM regions are coloured in blue and green respectively. In the reference films of P3HT-PC_71_BM, the distribution is representative of a bulk heterojunction as expected. We observe significant differences in the ternary bulk heterojunction films. The Zn_2_(ZnTCPP) MONs appear evenly distributed throughout film without breaking-up the P3HT-PC_71_BM heterojunction blend. On the other hand, the Cu_2_(CuTCPP) and Cu_2_(ZnTPyP) MONs seem to distribute themselves as larger aggregates in the film which breaks the P3HT-PC_71_BM charge percolation network.

The performance metrics of P3HT-PC_71_BM reference device and P3HT-Zn_2_(ZnTCPP)-PC_71_BM device stored outside the glovebox at room temperature for 1 month were tested again (ESI Sect. 12, Fig. S42, Tables S8 and S9). The PCE of the devices after 1 month MONs were found to be 1.44% with MONs and 0.76% without the MONs. This indicates that the presence of the nanosheets has no detrimental effect on the stability of the devices over time and continue to offer improved power conversion efficiencies as compared to the devices without nanosheets.

## Discussion

Here we have shown that the choice of organic ligand and metal ion can have a significant difference in device performance, either enhancing (Zn_2_(Zn-TCPP)), reducing (Cu_2_(CuTCPP) or having no-effect (Cu_2_(ZnTPyP)) compared to reference devices without nanosheets. We then explored three different possible underlying explanations for the differences observed, examining how structural differences affect optical absorption, energy level alignment, and morphology.

All three MON structures show complimentary light absorbance to those of P3HT and PC_71_BM, but some differences were observed for the different metal ion and ligand combinations. Zn_2_(ZnTCPP) has the highest molar extinction coefficient so will absorb more strongly than the other two MONs leading to a higher potential quantum efficiency. The position of Zn_2_(ZnTCPP)’s intense S band at 432 nm and two Q bands at 564 and 604 nm also overlap less than for the other nanosheets with absorption of P3HT. The Cu_2_(ZnTPyP) MON system has a relatively wide absorption range with two Q-bands and shows intermediary performance compared to Cu_2_(CuTCPP) which only has one Q band hence a comparatively narrow absorption range and a low molar extinction coefficient. We previously observed small improvements in the performances of devices made using amorphous polymers and ZnTCPP which was attributed to complimentary absorbance so it is possible that this accounts for some of the improvement observed^44^. However, P3HT has an much higher extinction coefficient than any of the MON systems so are expected to dwarf these small differences.

Previous work adding 2D materials to BHJ has attributed poor performance to the creation of traps due to misaligned energy levels. We therefore undertook detailed studies in order to understand energy level alignments within each of the systems. However, all the MONs have a workable energy level alignments within the error of the experiment. We have previously investigated other polymers where band gap alignment is less aligned than in these cases and still saw slightly improved device performance. We therefore conclude that band-gap alignment is also not the key criteria in explaining the differences in performance observed between these systems.

Our previous detailed studies on ZnTCPP with a variety of different donor polymers and fullerene acceptors indicate that the primary mechanism by which the nanosheets enhance performance is by acting as templates to increase the crystalline fraction of the surrounding semi-crystalline donor polymer. Raman and AFM studies indicate that Zn_2_(ZnTCPP) blend well with the P3HT: PC_71_BM to form reduced domain sizes compared to devices without nanosheets. In contrast, poor blending is observed in case of the Cu_2_(CuTCPP) MON system and Cu_2_(ZnTPyP). At a molecular level, this is surprising as you would expect Zn_2_(ZnTCPP) and Cu_2_(CuTCPP) to be more similar in surface properties than Cu_2_(ZnTPyP) which has an inverted PW structure. However, as all of the nanosheets were exfoliated under the same conditions, structural differences lead to variations in the thickness and lateral dimensions of the nanosheets. Cu_2_(CuTCPP) has the largest lateral dimensions 400–600 nm compared with 50–200 or 50–400 for the other two systems. Based on these results, we suggest that the large lateral dimensions of the nanosheets result in poor incorporation within the BHJ devices leading to poor device morphology and so reduced device performance. Further studies are needed to test this hypothesis and systematically explore the effect of nanosheet size on device performance.

## Conclusions

In conclusion, we took advantage of the modular nature of MONs to understand the effect of different components on the performance of archetypal OPV devices. The addition of Zn_2_(Zn-TCPP) resulted in a near doubling of the PCE whilst Cu_2_(Cu-TCPP) approximately halved PCE whilst Cu_2_(ZnTPyP) had no significant effect compared to reference devices without nanosheets. EQE and UV–vis data indicate that differences in light absorption between the MONs is small compared to absorption by P3HT, whilst UPS studies indicate that energy alignment within all the systems is within experimental error. Significant differences in device morphology were observed by AFM and Raman microscopy studies with monolayer Zn_2_(Zn-TCPP) favoring the formation of well-defined smaller grained sizes. Given the structural and electronic similarity of the three nanosheets, we conclude that nanosheet morphology plays a key role in determining device morphology and therefore performance. As a diverse class of nanomaterials, MONs offer enormous scope for further optimization of their electronic, optical, surface properties as well as their nanoscopic dimensions to further maximise performance. We anticipate that the insights from this systematic study will be useful in guiding the design of nanosheet based additives for use in OPV and other electronic devices.

## Supplementary Information

Supplementary Information.

## Data Availability

The Raman data are available through the file repository Zenodo at zenodo.org/records/12635101^65^. The other datasets generated during and/or analysed during the current study are available from the corresponding authors on reasonable request.
