# Supplementary material for: Tuning the morphology and energy levels in organic solar cells with metal–organic framework nanosheets
Source: Sci Rep. 2024 Nov 28;14:29559. doi: 10.1038/s41598-024-80007-y (PMC11605120; doi:10.1038/s41598-024-80007-y)
Supplement: Supplementary file 1 — Supplementary Information. [file 41598_2024_80007_MOESM1_ESM.pdf]

Tuning the morphology and energy levels in organic solar cells with metal-organic framework nanosheets

Electronic Supplementary Information

Kezia Sasitharan [a,b*], Johannes Frisch [c,d], Jaroslav Kulicek [b], Ahmed Iraqi [a], David G. Lidzey [e], Marcus Bär [c, d ,f, g], Bohuslav Rezek [b] and Jonathan A. Foster [a*]

[a] Department of Chemistry, The University of Sheffield, Dainton Building, Brook Hill, S3 7HF, Sheffield, UK.

[b] Centre for Advanced Photovoltaics, Faculty of Electrical Engineering, Czech Technical University in Prague, 16000 Czech Republic

[c] Interface Design, Helmholtz-Zentrum Berlin für Materialien und Energie GmbH (HZB), 12489 Berlin, Germany

[d] Energy Materials In - Situ Laboratory Berlin (EMIL), HZB, 12489 Berlin, Germany

[e] Department of Physics and Astronomy, The University of Sheffield, Hicks Building, Hounsfield Road, S3 7RH, Sheffield, UK

[f] Department of Chemistry and Pharmacy, Friedrich-Alexander-Universität Erlangen-Nürnberg (FAU), 91054 Erlangen, Germany

[g] Department X-ray Spectroscopy at Interfaces of Thin Films, Helmholtz Institute Erlangen-Nürnberg for Renewable Energy (HI ERN), 12489 Berlin, Germany

Contents

1. **General Details**
2. **Synthetic procedures**
3. **XRD**
4. **UV-Vis absorption spectroscopy**
5. **Atomic force microscopy imaging**
6. **Device fabrication**
7. **Device testing**
8. **Bilayer devices**
9. **Photoemission studies**
10. **AFM – morphology of thin films**
11. **Raman Microscopy and true component analysis**
12. **References**

1 General Details

1.1 Materials

Commercial solvents and reagents were used without further purification. Synthesis of organic ligands was carried out in dry glassware with a nitrogen overpressure. Solvothermal synthesis of metal-organic frameworks was undertaken using borosilicate vials with Teflon faced rubber lined caps.

1.2 Analytical Procedures

NMR spectra were recorded on a Bruker Advance DPX 400 spectrometer. Chemical shifts for ^1H are reported in ppm on the δ scale; ^1H chemical shifts were referenced to the residual solvent peak. All coupling constants are reported in Hz. Mass spectra were collected using a Bruker Reflex III MALDI-TOF spectrometer. Elemental analyses were obtained on a vario MICRO CHNS elemental analyzer equipped with a thermal conductivity detector. X-Ray powder diffraction patterns were collected using a Bruker D8 Advance powder diffractometer equipped with a copper K_α source (λ=1.5418 Å) operating at 40 kV and 40 mA. The instrument was fitted with an energy-dispersive LYNXEYE detector. Measurements were carried out using a fixed goniometer stage with a rotating flat plate sample holder. IR spectroscopy was performed on a Perkin-Elmer Pyris TGA from 30-600°C at 10°C min^{-1}, under a 20 mL min^{-1} nitrogen flow. UV-Vis absorption spectra were collected on a Cary 5000 UV-Vis-NIR instrument using a 1 cm internal length quartz cuvette.

Nanoscopic characterisation was performed using a Bruker Multimode 5 AFM, operating in soft-tapping mode under ambient conditions. Bruker OTESPA-R3 cantilever were used, with a drive amplitude and nominal resonance frequency of 20.4 mV and 290 kHz, respectively. Images were processed using standard techniques with free Gwyddion software.

XPS and UPS measurements were performed in the Energy Materials In-Situ Laboratory Berlin (EMIL) part of the Helmholtz-Zentrum Berlin für Materialien und Energie GmbH (HZB). The samples were prepared and mounted in a N_2-purged glovebox directly attached to the (ultra-high vacuum) UHV-backbone of EMIL. UPS and XPS measurements were performed using the surface analysis system (base pressure < 5 × 10^{-9} mbar) employing a non-monochromatized PREVAC gas discharge UV excitation source and a RS40B1 Mg K_α/Al K_α twin anode X-ray source, respectively, and a ScientaOmicron Argus CU electron analyzer. The samples were

studied with He I (21.2 eV) and Mg K_{α} (1253.6 eV), Al K[illegible] (1486.6 eV) excitation. The UPS and XPS energy scale was calibrated using a clean Au foil, setting the Fermi level to 0.0 eV binding energy (BE) and the Au $4f_{7/2}$ peak to a BE of 84.0 eV, respectively. Pass energies for the measurements were set to 50 and 20 eV for the XPS survey and core level detail spectra, respectively, and to 2 eV for the UPS spectra of the secondary electron cut off (SECO) and the valence band (VB) region. For the SECO measurements, a BIAS of -10.0 V was applied to the samples.

Raman maps and spectra were recorded on the WITec alpha 300 RAS in a reflection mode using the integrated spectrometer (fitted with a CCD detector) and fiber-coupled 532 nm laser for excitation. A 100x objective was used to focus the 532 nm excitation laser on the sample surface and collect the signal. Raman maps were recorded in an area of 25x25µm with a scan speed of 30 seconds/line and an integration time of 0.2s. Software Control 5.2 and Project 5.2 were used to collect and evaluate data.

2 Synthetic procedures

2.1 Synthesis of *meso-tetracarboxyphenyl porphyrin* (TCPP)

10 mL of pyrrole (98%, light brown solution) was distilled under vacuum at 80°C to give 5 mL of a clear solution. 4-formylbenzoic acid (3.0 g, 20 mmol) was dissolved in propionic acid (100 mL) and freshly distilled pyrrole (1.4 mL, 20 mmol) was added by syringe. The solution immediately darkened and was refluxed for 15 hours. The mixture was chilled in a fridge for 10 hours before collection of the solid by vacuum filtration. The solid was washed with hot water (2 × 20 mL) and dried under vacuum to give the product as a black powder (3.23 g, 4.0 mmol, 80 %). Elemental Analysis (%) calculated for $C_{48}H_{30}N_4O_8$: Expected: C, 72.91; H, 3.82; N, 7.09. Found: C, 72.95; H, 4.08; N, 6.82

λ_{max} (nm): 420 (π-π^*)

^{1}H-NMR (d_6-DMSO) δ/ppm: 8.85 (8H, s, β-pyrrolic H), 8.38 (8H, d, *J* = 8.0, Ar-H), 8.32 (8H, d, *J* = 8.0, Ar-H), -2.95 (2H, s, internal pyrrole NH).

MALDI-TOF: m/z 791.2 ($[MH]^+$)

2.2 Synthesis of *tetrapyridyl porphyrin* (TPyP)

10 mL of pyrrole (98%, light brown solution) was distilled under vacuum at 80°C to give 5ml of a clear solution. 4-pyridine-carboxaldehyde (3.16 mL, 3.35 mmol) was dispersed in propionic acid (75 ml). This mixture was heated to 100°C under reflux. A mixture of pyrrole (2.32 ml, 3.34 mmol) and propionic acid (10 ml, 0.13 mmol) was added slowly and the resulting mixture is heated to 141°C for 24 hours. The mixture was chilled in the fridge for about 12 hours before adding hot water to the solution to force the TPyP out of solution. The solid was collected by vacuum filtration and washed with hot water (4 × 20ml) and dried under vacuum to give the product as a black powder.

λ_{max} (nm): 418 (π-π^*)

^{1}H-NMR ($CDCl_3$) δ/ppm: 9.04 (8H, d, 2,6 pyridyl), 8.8 (8H, m, pyrrole), 8.18 (8H, m, 3,5 pyridyl), -2.95 (2H, s, internal pyrrole NH).

2.3 Metallation of TPyP with Zn

Zinc (II) was inserted into the porphyrin monomers by standard methods. An excess of Zinc Nitrate (1.1 mmol) was added to 0.63 mmol of TPyP in 50 mL DMF. The solution was refluxed for 1 hour, after which a small sample fo the reaction mixture was extracted and analysed by using UV-Vis spectroscopy. The spectrum indicated that the metal insertion was complete with the four Q bands of the free base collapsing into two. The DMF was rotavaped off and the ZnTPyP was washed with hot water (60°C, 2 × 20mL).

λ_{max} (nm): 422 (π-π^*)

2.4 Synthesis of bulk Zn_2(Zn-TCPP)(DMF)

H_2TCPP (7.9 mg, 0.03 mmol), $Zn(NO_3)_2.3H_2O$ (8.9 mg, 0.09 mmol), DMF (1.5 mL) and ethanol (0.5 mL) were mixed at room temperature and heated at 80°C for 24 hours. Purple crystals were collected by centrifugation at 4500 RPM for 10 minutes and repeatedly washed in ethanol until the supernatant became clear (Yield 87%)

Elemental analysis $C_{48}H_{24}N_4O_8Zn_3(H_2O)_4(DMF)_3$: Expected C, 53.57; H, 4.50; N, 8.33 Found C, 52.76; H, 4.77; N, 8.88.

A slight excess of Zn around the edge of the MOF and possible nitrate counter ions could be the reason for lower carbon content and higher nitrogen.

2.5 Synthesis of bulk Cu_2(Cu-TCPP)(DMF)

H_2TCPP (23.7 mg, 5 mmol), $Cu(NO_3)_2.3H_2O$ (21.6 mg, 15 mmol), DMF (4.5 mL) and ethanol (1.5 mL) were mixed at room temperature and heated at 80°C for 24 hours. Purple powder was collected by centrifugation at 4500 RPM for 10 minutes and repeatedly washed in ethanol until the supernatant became clear (Yield 83.8%)

Elemental analysis $C_{48}H_{24}N_4O_8Cu_3(H_2O)_4(DMF)_3$: Expected C, 54.05; H, 4.22; N, 7.74; Found C, 53.45; H, 4.44; N, 8.04

2.7 Synthesis of bulk Cu_2(Zn-TPyP)(DMF)

ZnTPyP (7.9 mg, 0.03 mmol), $Cu(NO_3)_2.3H_2O$ (8.9 mg, 0.09 mmol), DMF (1.5 mL) and ethanol (0.5 mL) were mixed at room temperature and heated at 80°C for 24 hours. Black powdery product was collected by centrifugation at 4500 RPM for 10 minutes and repeatedly washed in ethanol until the supernatant became clear (Yield 74%)

Elemental analysis $C_{49}H_{43}N_8O_8Cu_2Zn(EtOH)_3(DMF)_2$: Expected C, 54.57; H, 4.75; N, 11.57; Found C, 54.15; H, 4.64; N, 10.04

2.8 Exfoliation of MOF into MONs

5 mg of MOF was added to a 12 mL glass vial along with 6 mL of the desired solvent. The sample was mixed in a vortex mixer for 30 seconds to disperse the sediment. The samples were sonicated using a Fisherbrand Elmasonic P 30H ultrasonic bath (2.75 L, 380/350 W, UNSPSC 42281712) filled with water. Samples were sonicated for 60 min at a frequency of 80 kHz with 100% power and the temperature was thermostatically maintained at 16-20°C using a steel cooling coil. Sonication was applied using a sweep mode and samples were rotated through the water using an overhead stirrer to minimise variation due to ultrasound "hot-spots". Following sonication, the vials were transferred to centrifuge tubes and centrifuged at 1500 RPM for 10 minutes to remove non-exfoliated particles.

2.9 Sample preparation for UPS and XPS analysis

Pre-patterned ITO Glass substrates (20 mm x 15 mm) of 20 Ohm/square resistance (Ossila) were cleaned via hellmenex (1-3vol%) in boiling DI water (10-minute sonication), followed by further washing in boiling DI water (10-minute sonication) and finally 5-10 minutes sonication in isopropyl alcohol**.** The substrates were dried with a nitrogen gun and placed in a UV-Ozone cleaner for 10 minutes to ensure removal of any surface particulates. PEDOT:PSS solution (Al4083, Ossila) was brought to room temperature, filtered through a 0.45 µm PVDF filter into an amber vial and dynamically spin-coated (45 µL) onto room-temp substrates at 6000 rpm, to achieve a film ~25 nm. The PEDOT:PSS coated substrates were placed on a hotplate at 110 °C for 15 minutes. This was followed by further annealing in the glovebox at 110 °C for 15 minutes to remove any surface moisture. After 15 minutes annealing in the glovebox, the ITO/PEDOT:PSS samples were cooled to room temperature before active layer deposition. P3HT (10mg/mL) was dissolved in chlorobenzene solvent. After heating at 60 °C for 10 minutes followed by cooling towards room temperature, the solution was filtered through a 0.45 µm PTFE filter. The solutions were then spin-cast onto the ITO/PEDOT:PSS substrate at 1000 rpm under a nitrogen atmosphere in a glove box, forming films of ∼150 nm as determined by Dektak®. For the MONs, suspensions of 5mg/mL from ethanol were spin-coated at 500 rpm for 30 seconds. Since MONs are anisotropic materials that are spin-coated from suspension rather than solution, the resulting film is made of MON “islands” on top of the PEDOT:PSS substrate.

3. XRD of MOF and MONs

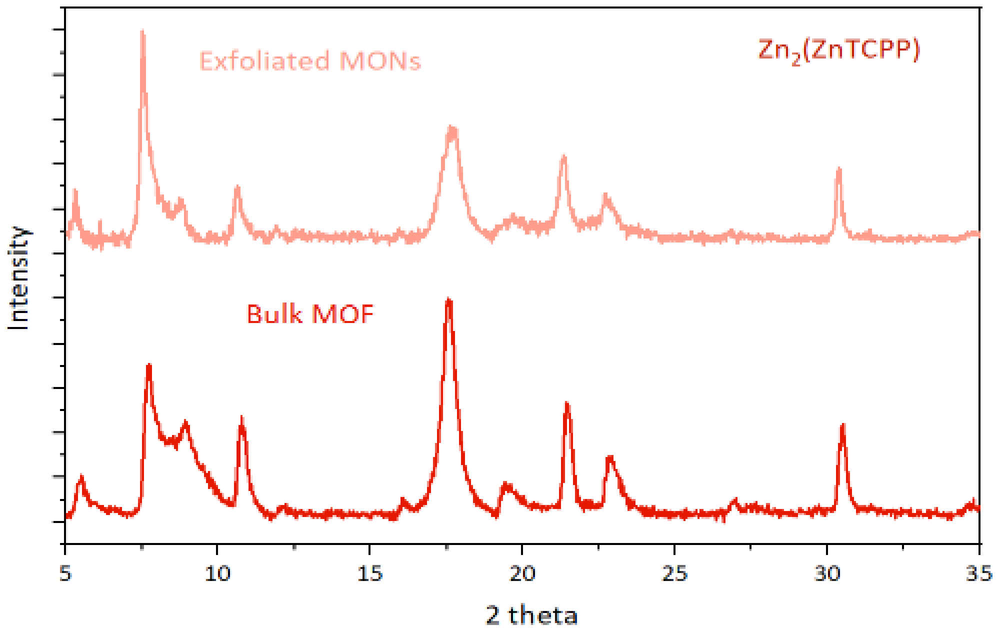

Figure S1. PXRD patterns obtained from Zn_2(ZnTCPP) bulk MOF compared to the exfoliated MONs.

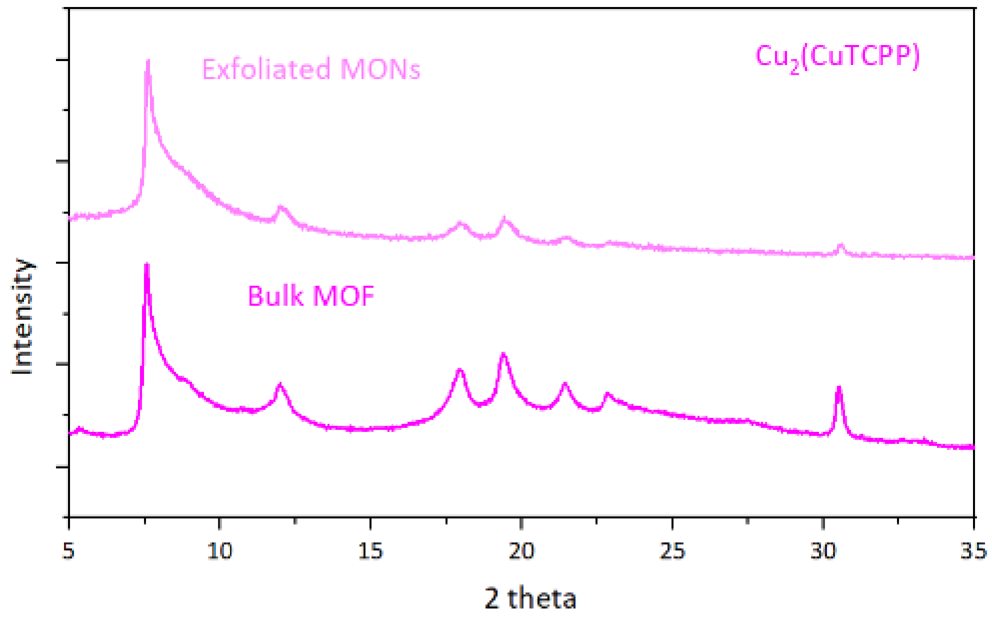

Figure S2. PXRD patterns obtained from Cu_2(CuTCPP) bulk MOF compared to the exfoliated MONs.

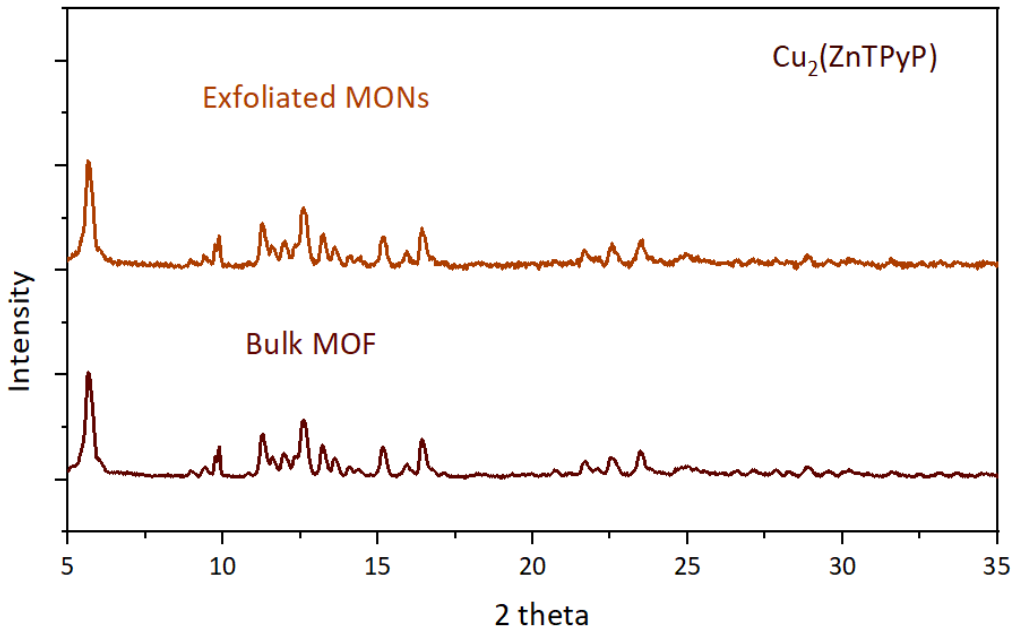

Figure S3. PXRD patterns obtained from Cu_2(ZnTPyP) bulk MOF compared to the exfoliated MONs.

4. Molar extinction coefficients

4.1 Zn_2(ZnTCPP) MONs

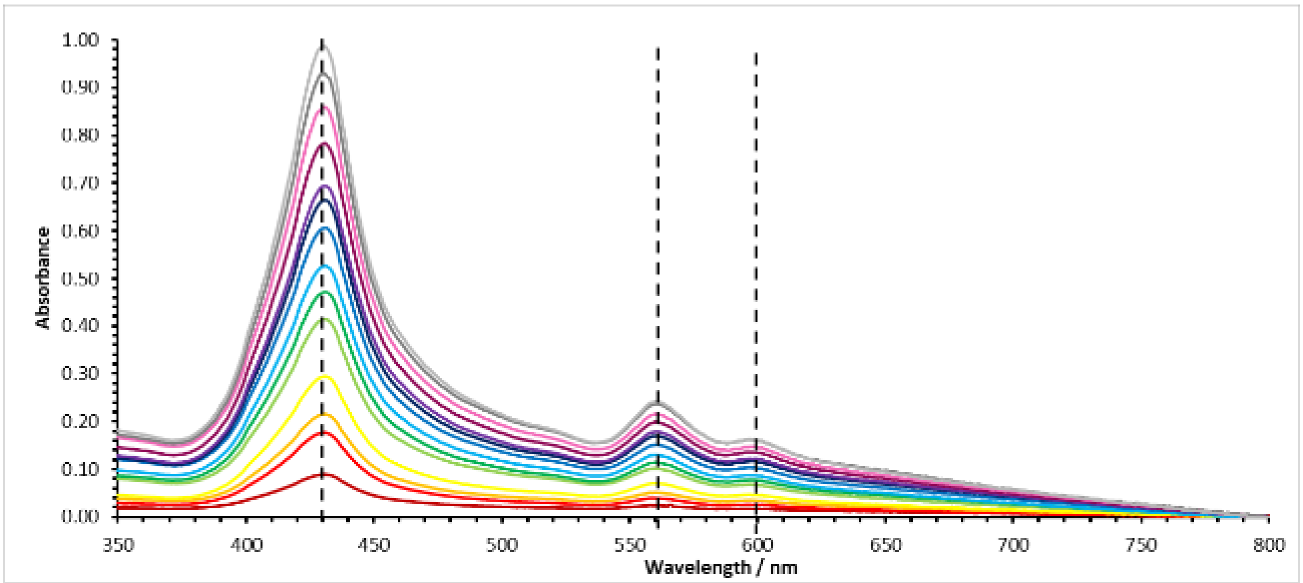

Figure S4. UV-Vis absorption stack plots for Zn_2(ZnTCPP) exfoliated in ethanol

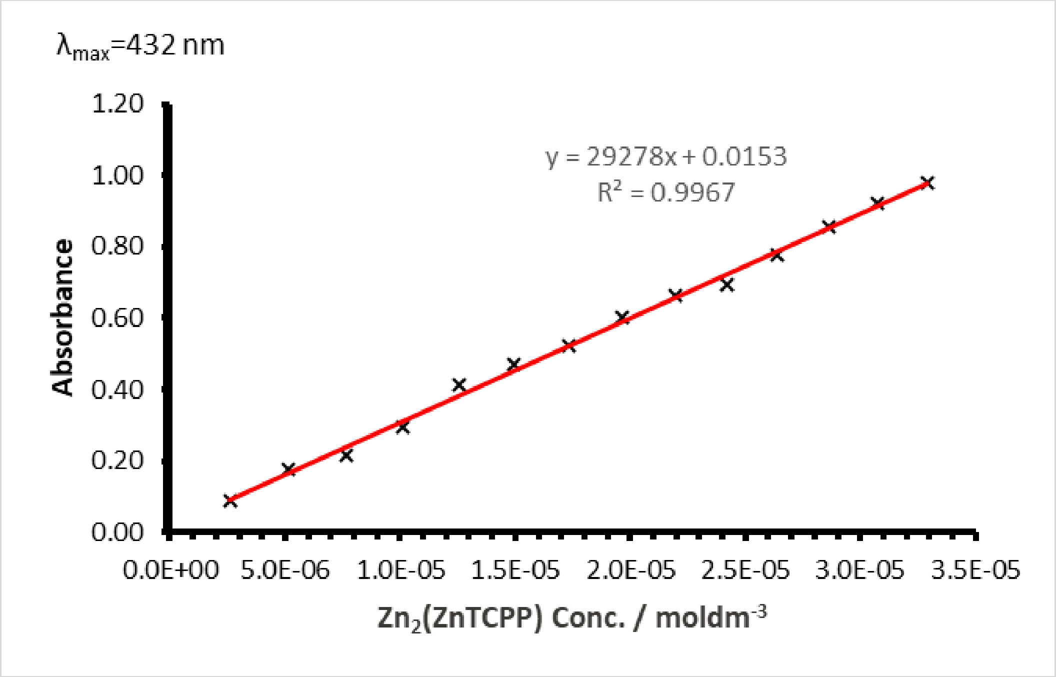

Figure S4a. Extinction coefficient calculation graphs for Zn_2(ZnTCPP) exfoliated in ethanol at the indicated wavelengths and concentrations.

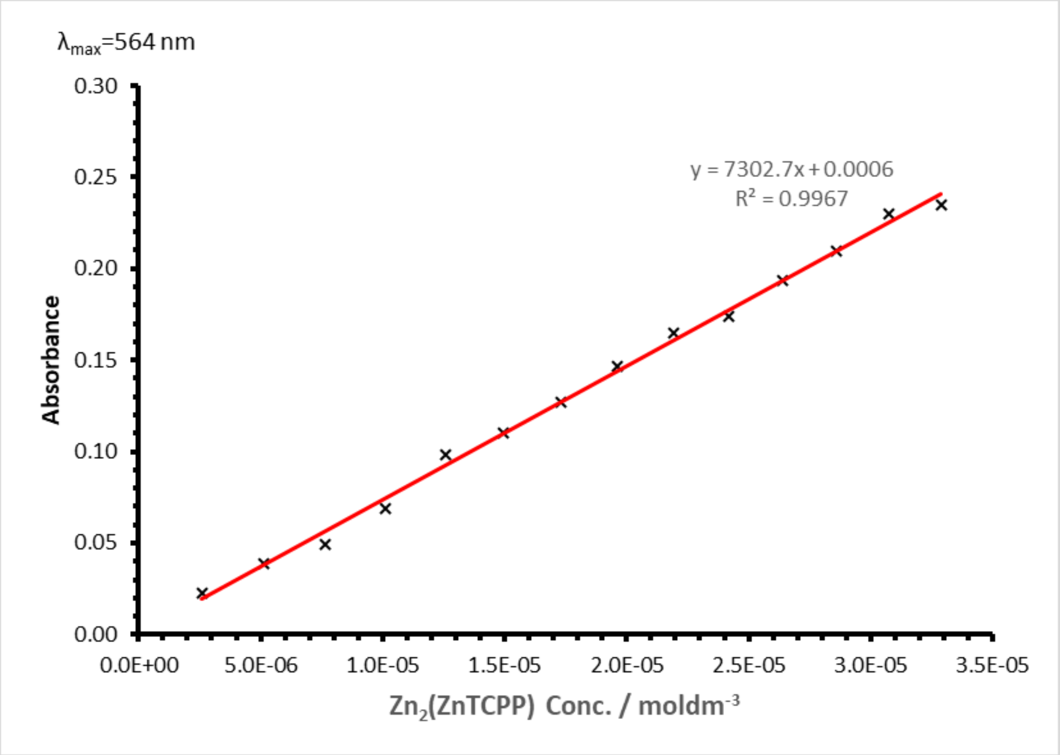

Figure S4b. Extinction coefficient calculation graphs for Zn_2(ZnTCPP) exfoliated in ethanol at the indicated wavelengths and concentrations.

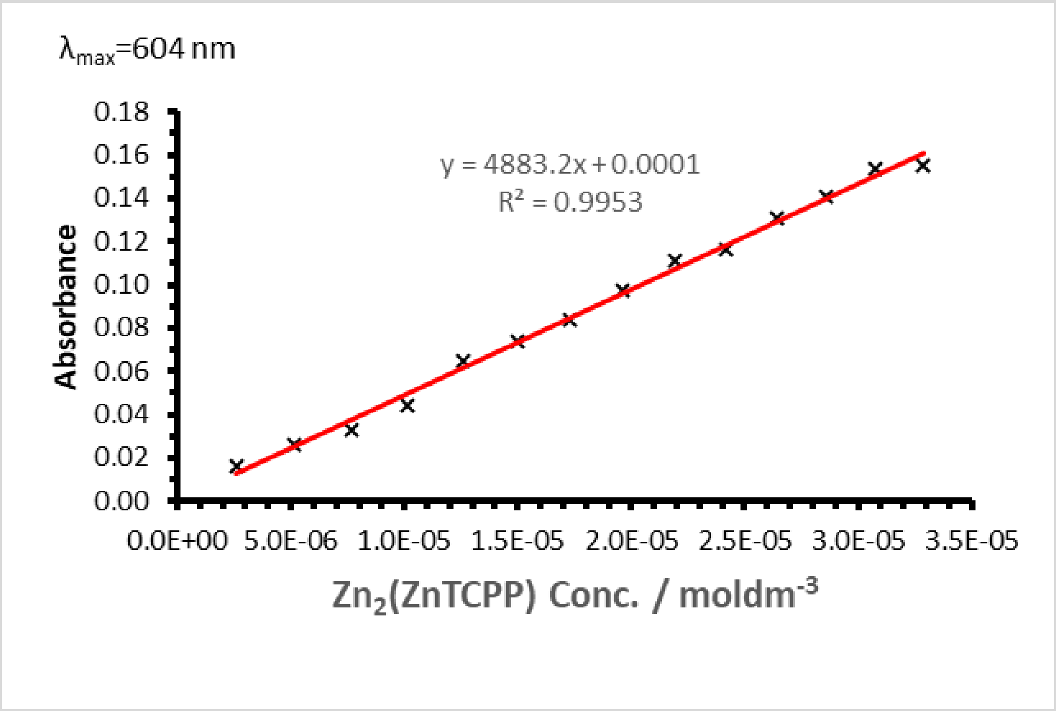

Figure S4c. Extinction coefficient calculation graphs for Zn_2(ZnTCPP) exfoliated in ethanol at the indicated wavelengths and concentrations.

4.2 Cu_2(CuTCPP) MONs

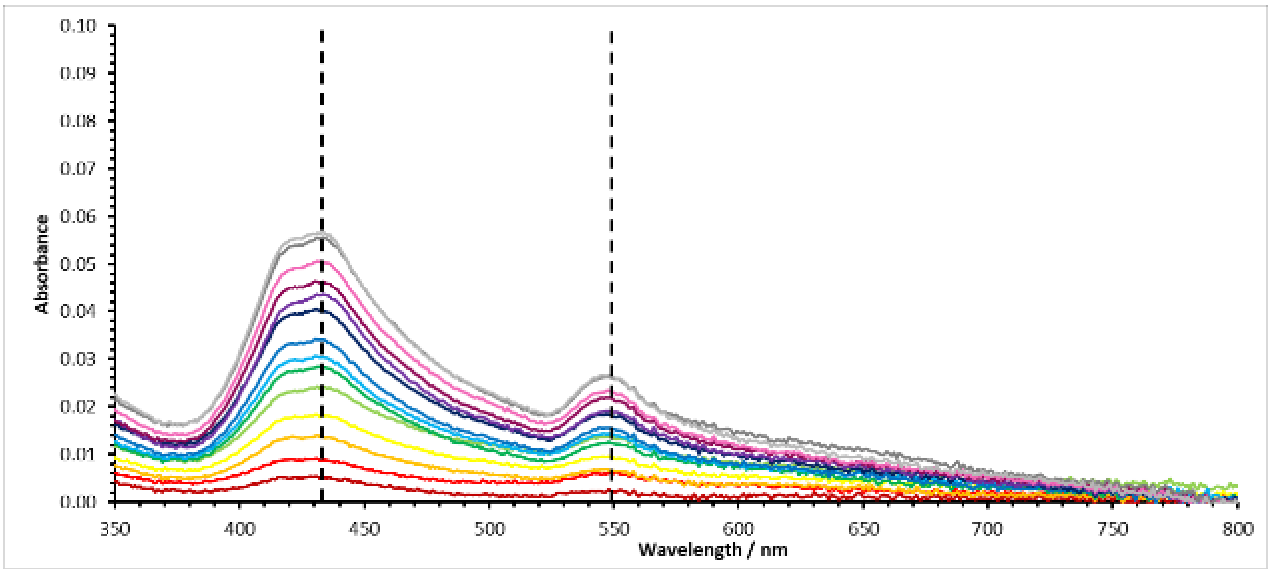

Figure S5. UV-Vis absorption stack plots for Cu_2(CuTCPP) exfoliated in ethanol.

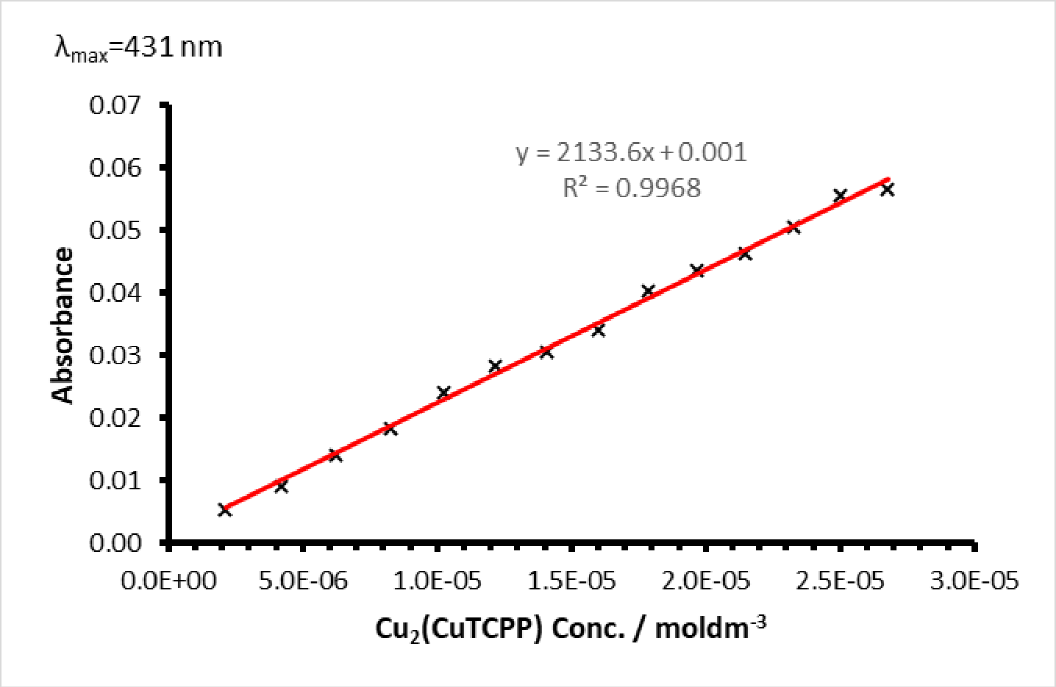

Figure S5a. Extinction coefficient calculation graphs for Cu_2(CuTCPP) exfoliated in ethanol at the indicated wavelengths and concentrations.

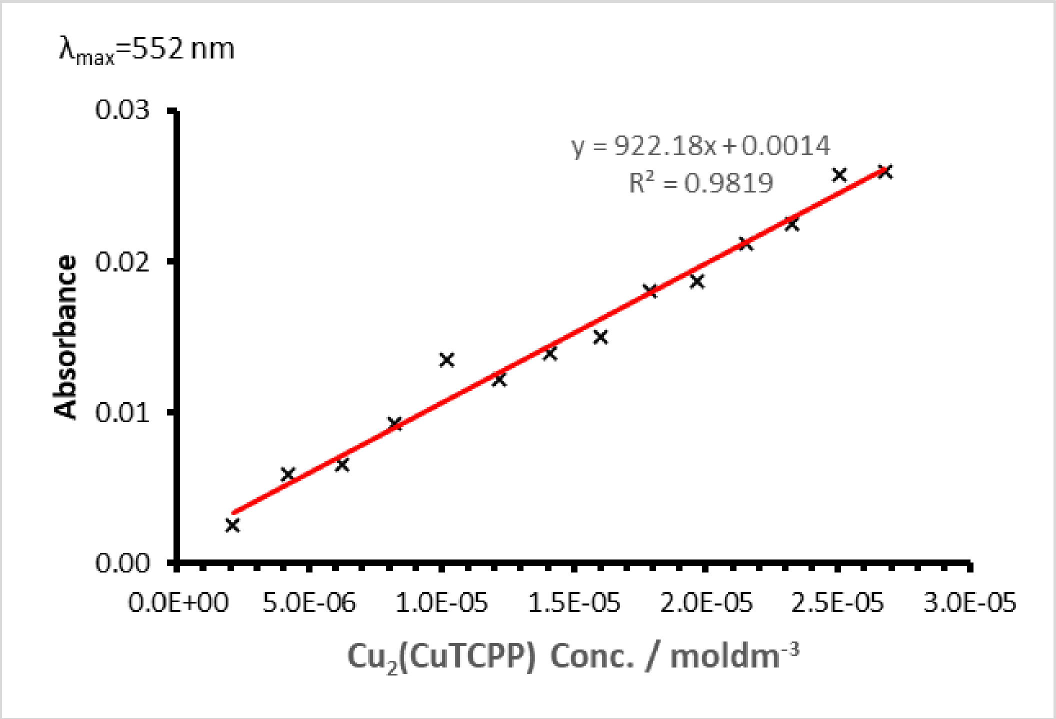

Figure S5b. Extinction coefficient calculation graphs for Cu_2(CuTCPP) exfoliated in ethanol at the indicated wavelengths and concentrations.

4.3 Cu_2(ZnTPyP) MONs

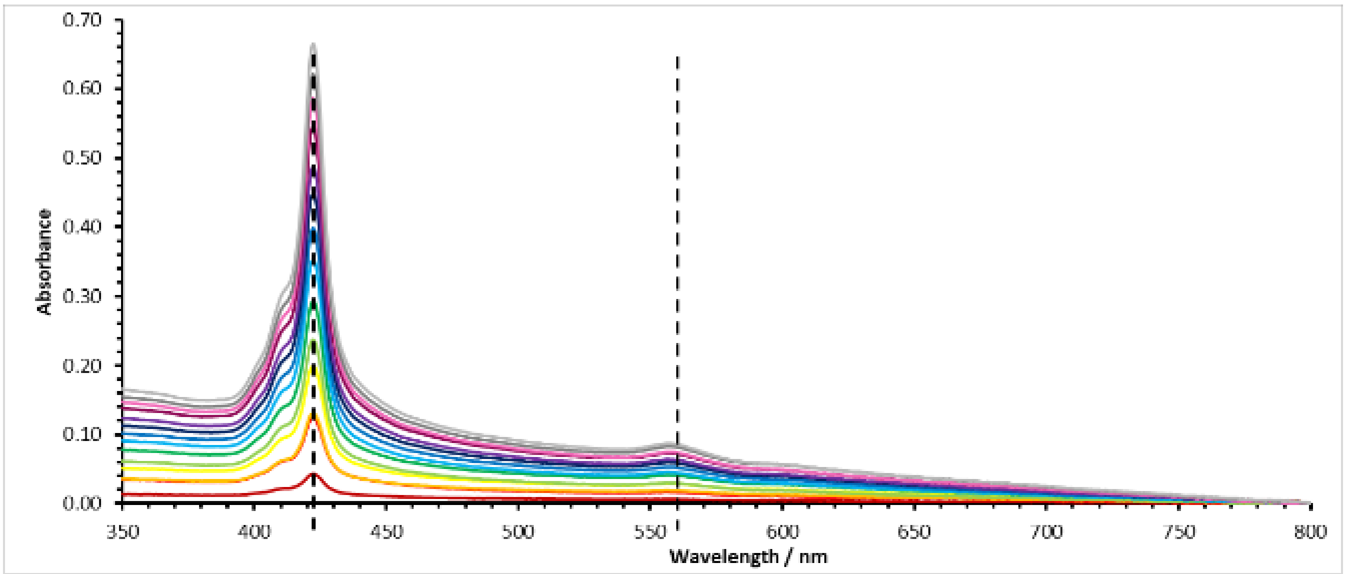

Figure S6. UV-Vis absorption stack plots for Cu_2(CuTPyP) exfoliated in ethanol

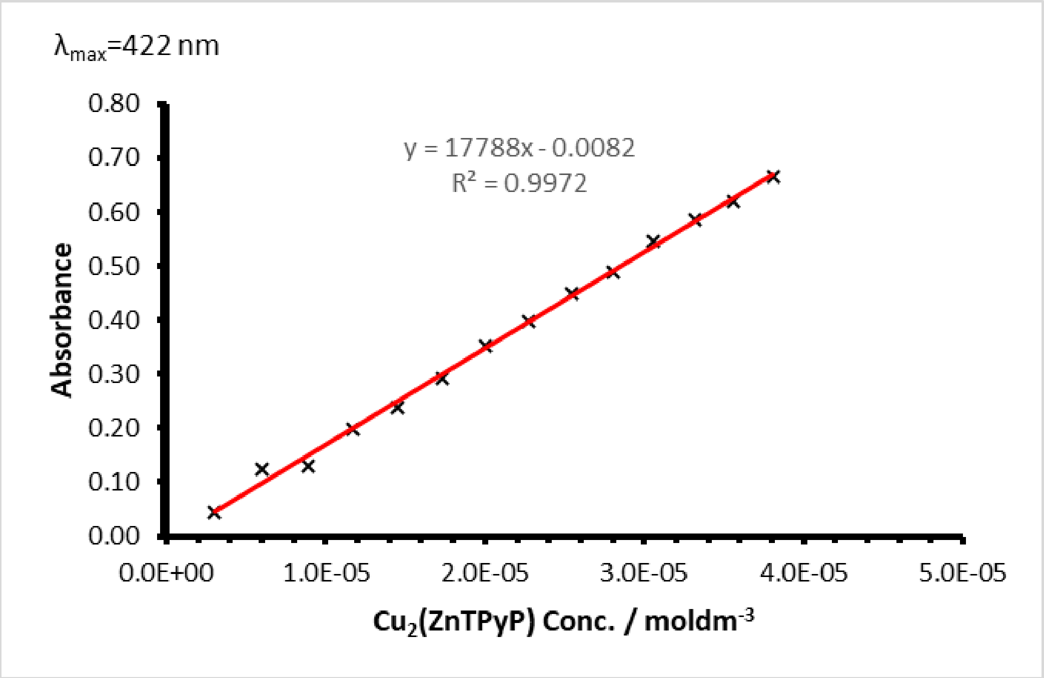

Figure S6a. Extinction coefficient calculation graphs for Cu_2(CuTPyP) exfoliated in ethanol at the indicated wavelengths and concentrations.

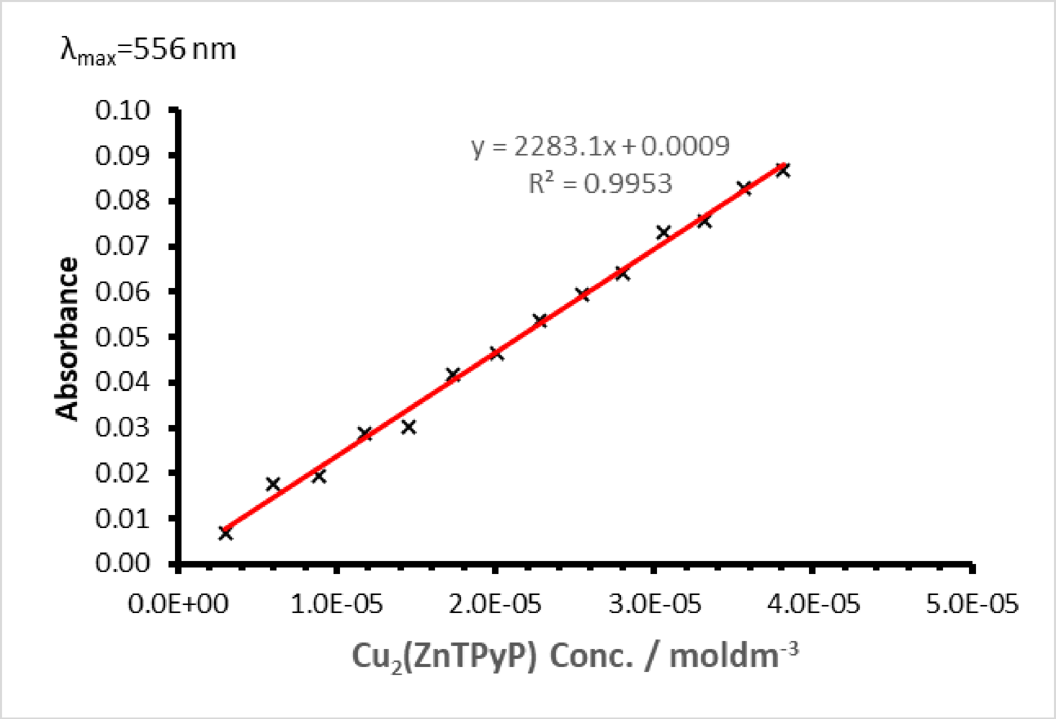

Figure S6b. Extinction coefficient calculation graphs for Cu_2(CuTPyP) exfoliated in ethanol at the indicated wavelengths and concentrations.

5. AFM imaging of various MONs

5.1 $Zn_2(ZnTCPP)$ MONs

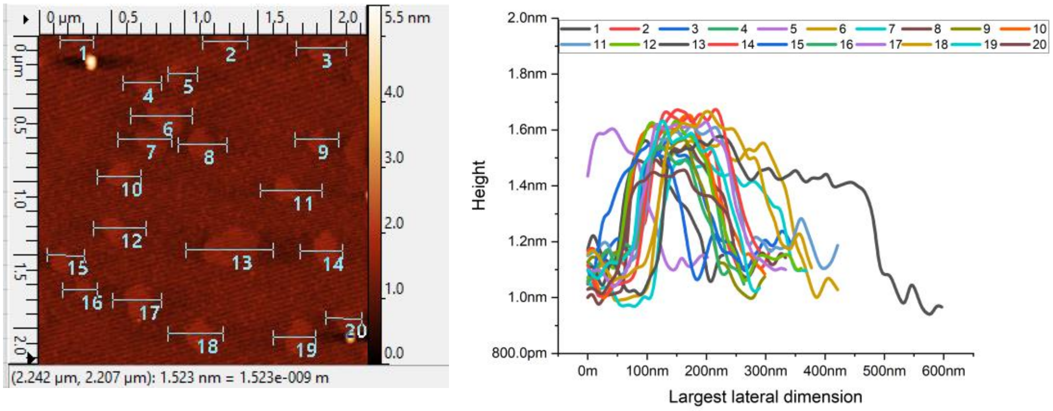

Figure S7. Atomic force microscopy images of exfoliated nanosheets of $Zn_2(ZnTCPP)$ and associated height profiles.

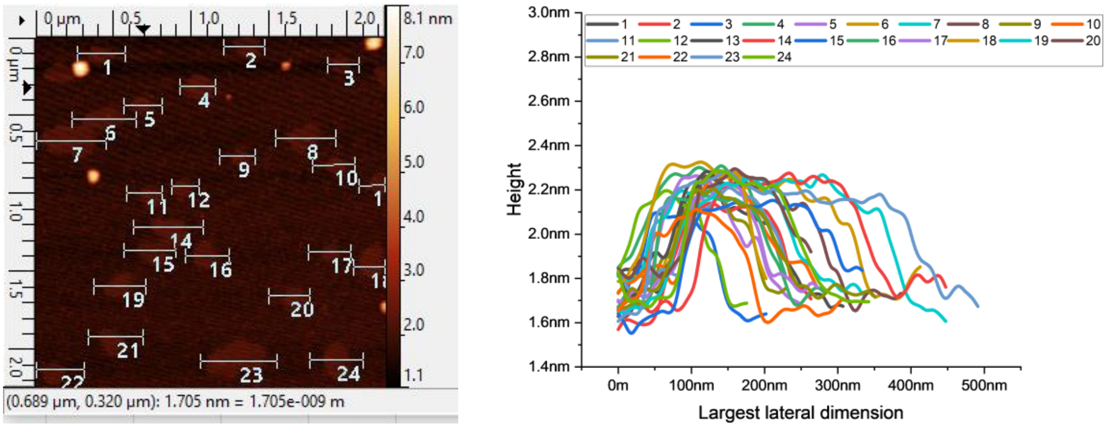

Figure S8. Atomic force microscopy images of exfoliated nanosheets of $Zn_2(ZnTCPP)$ and associated height profiles.

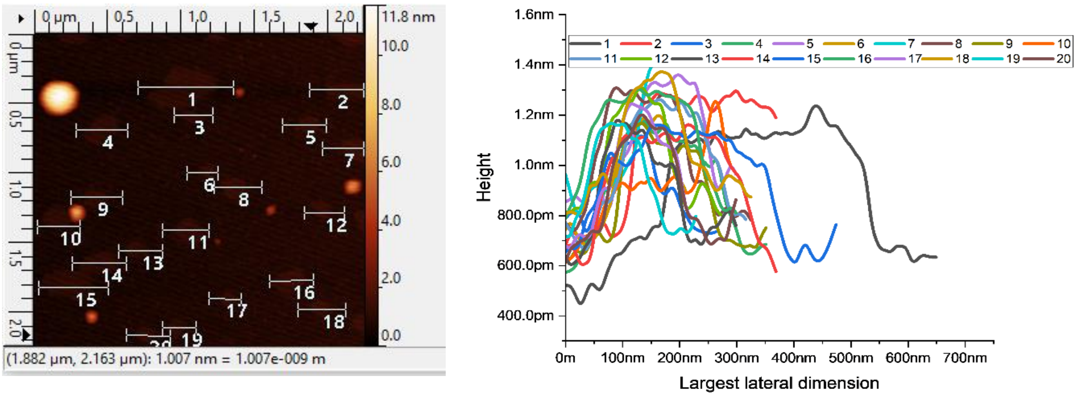

Figure S9. Atomic force microscopy images of exfoliated nanosheets of $Zn_2(ZnTCPP)$ and associated height profiles.

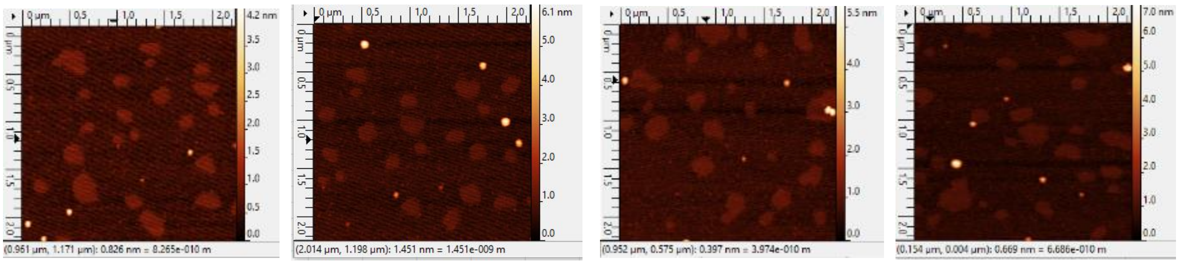

Figure S10. Additional Atomic force microscopy images of exfoliated nanosheets of $Zn_2(ZnTCPP)$ MONs.

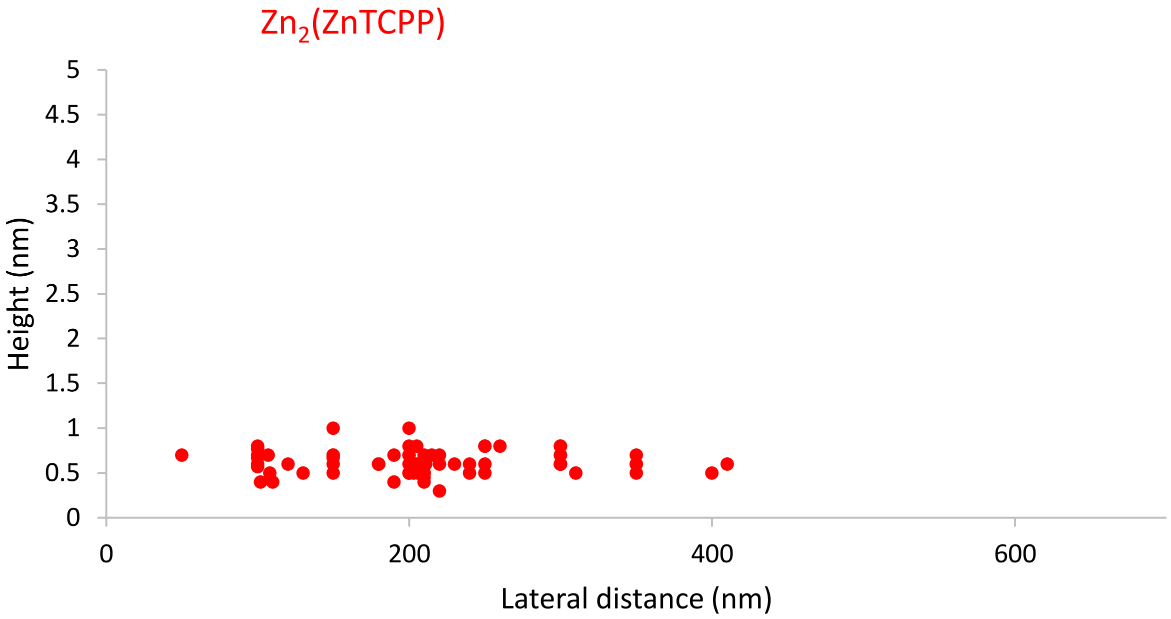

Figure S11. Size distribution scatter plot of $Zn_2(ZnTCPP)$ nanosheets derived from the above images (S7-S10). The nanosheets consist of predominantly monolayer nanosheets (~1nm).

5.2 $Cu_2(CuTCPP)$ MONs

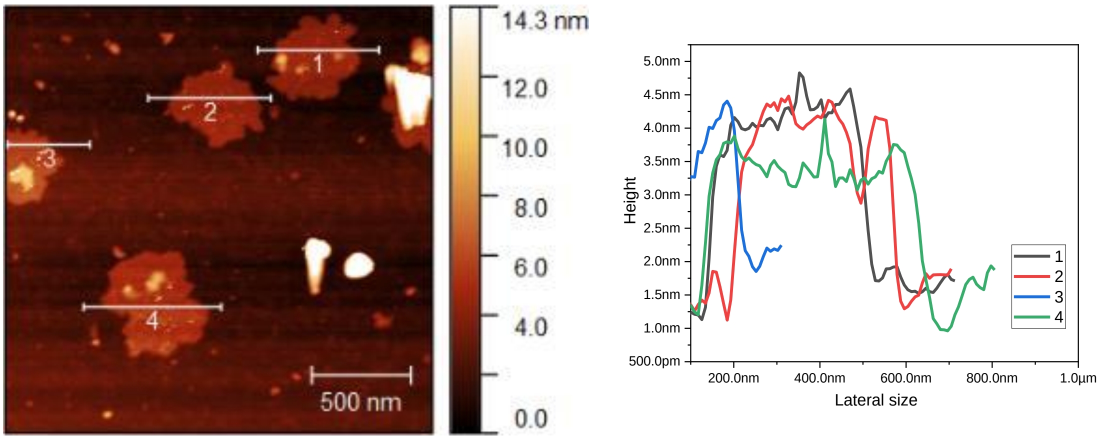

Figure S12 Atomic force microscopy images of exfoliated nanosheets of $Cu_2(CuTCPP)$ and associated height profiles.

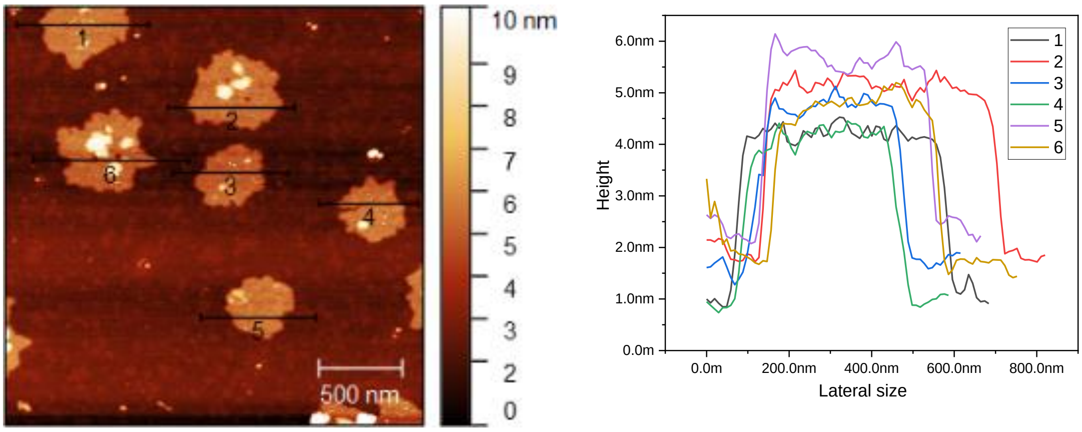

Figure S13 Atomic force microscopy images of exfoliated nanosheets of $Cu_2(CuTCPP)$ and associated height profiles.

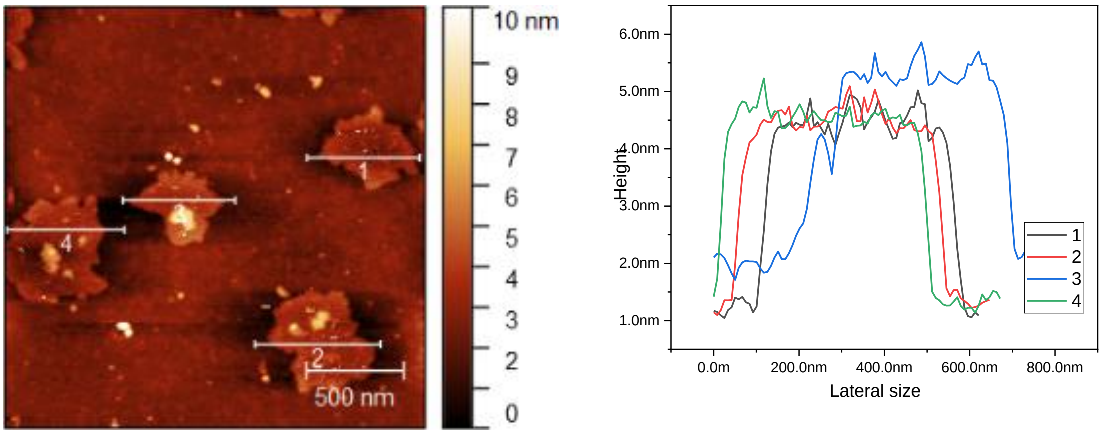

Figure S14 Atomic force microscopy images of exfoliated nanosheets of $Cu_2(CuTCPP)$ and associated height profiles.

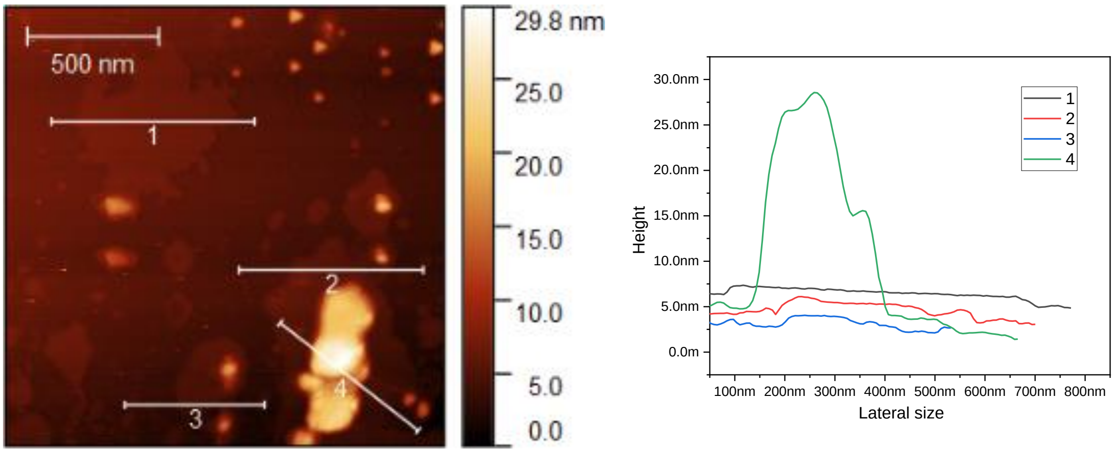

Figure S15. Atomic force microscopy images of exfoliated nanosheets of $Cu_2(CuTCPP)$ and associated height profiles.

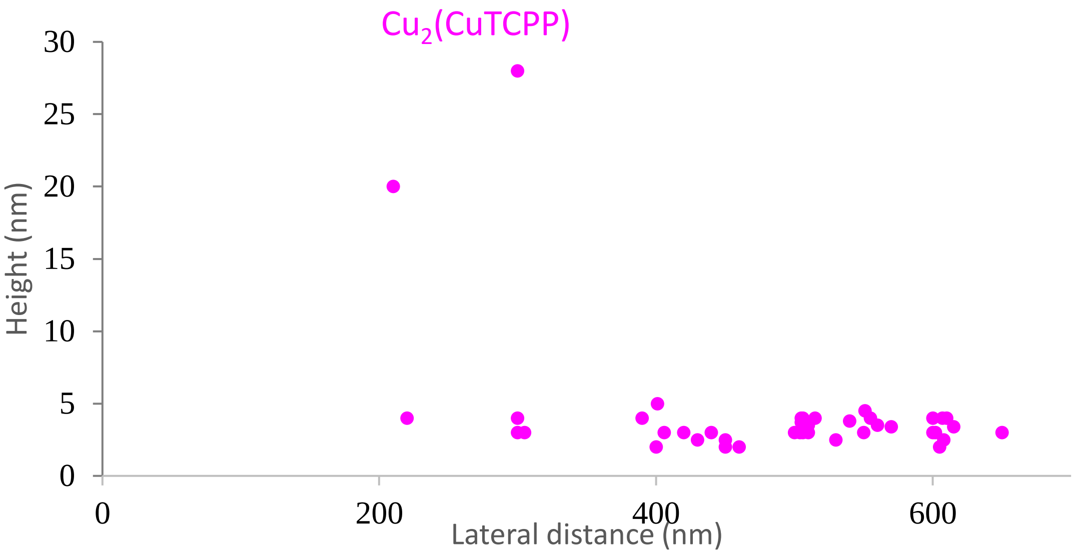

Figure S16. Size distribution scatter plot of $Cu_2(CuTCPP)$ nanosheets derived from the above images (S11-S15).

5.3 Cu_2(ZnTPyP) MONs

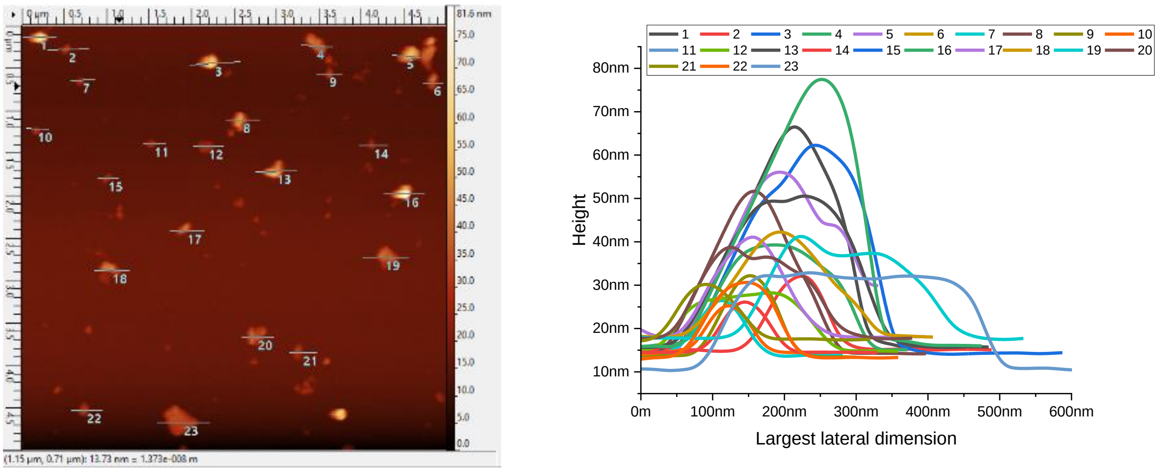

Figure S17. Atomic force microscopy images of exfoliated nanosheets of Cu_2(ZnTPyP) and associated height profiles.

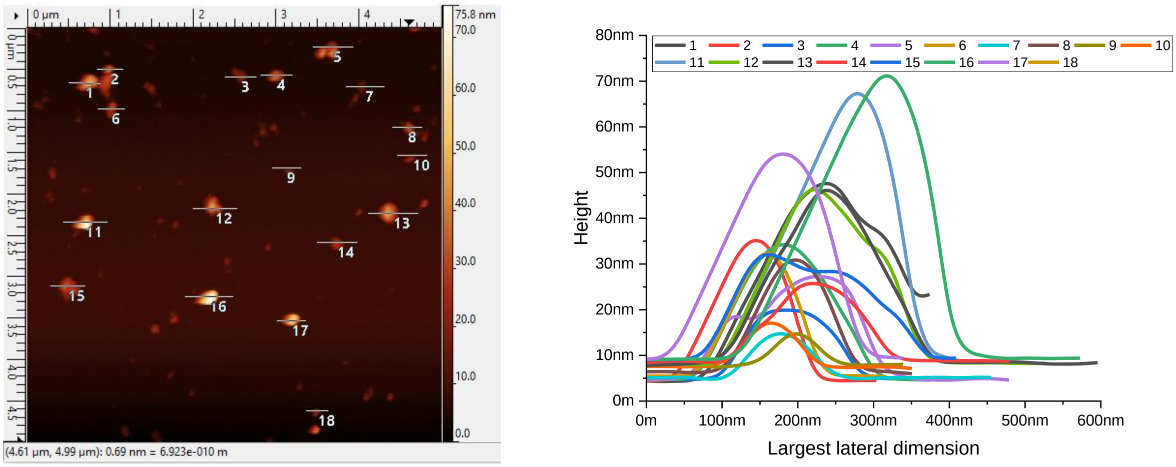

Figure S18. Atomic force microscopy images of exfoliated nanosheets of Cu_2(ZnTPyP) and associated height profiles.

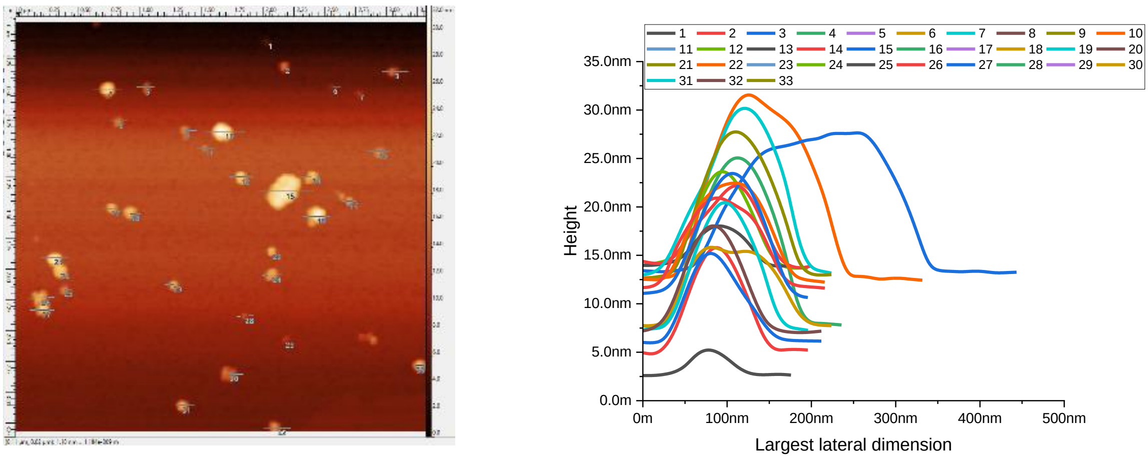

Figure S19. Atomic force microscopy images of exfoliated nanosheets of $Cu_2(ZnTPyP)$ and associated height profiles.

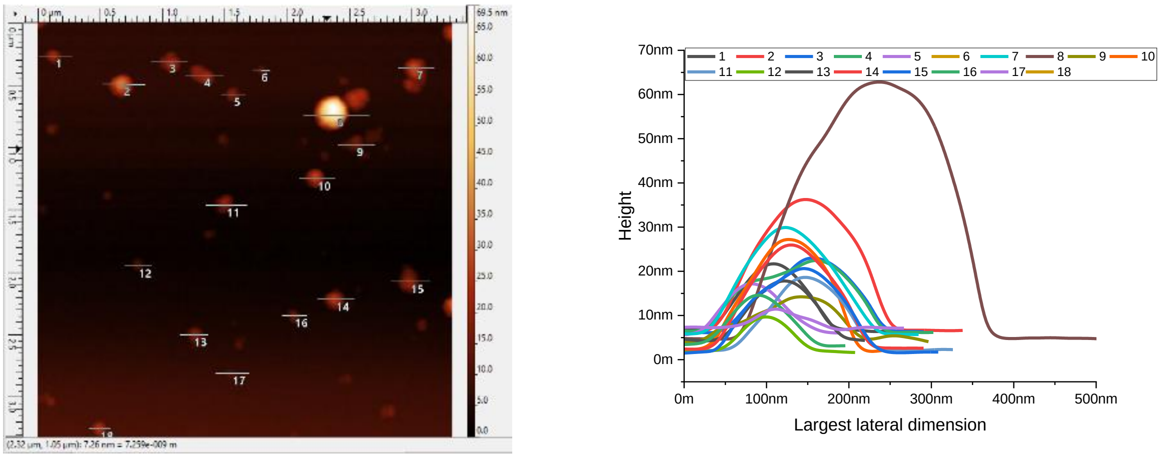

Figure S20. Atomic force microscopy images of exfoliated nanosheets of $Cu_2(ZnTPyP)$ and associated height profiles.

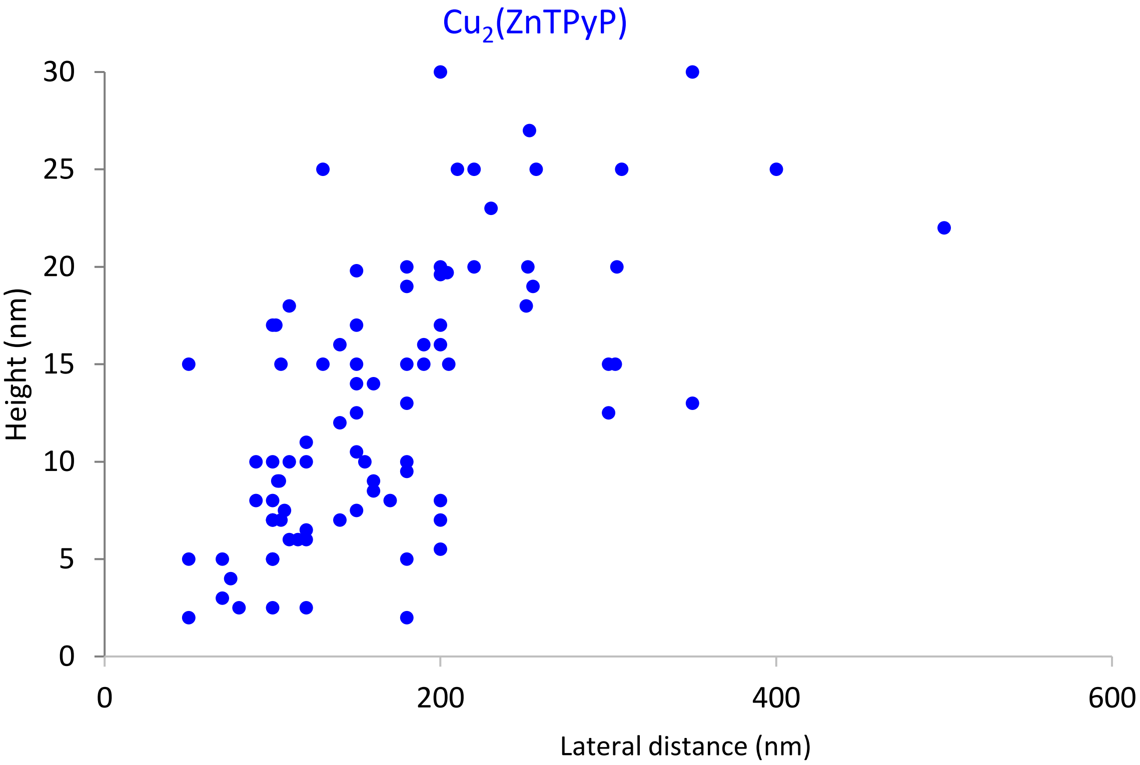

Figure S21. Size distribution scatter plot of $Cu_2(ZnTPyP)$ nanosheets derived from the above images (S17-S20).

6. Fabrication of devices

6.1 Preparation of substrates

Pre-patterned ITO Glass substrates (20 mm x 15 mm) of 20 Ohm/square resistance (Ossila) were cleaned via hellmenex (1-3vol%) in boiling DI water (10-minute sonication), followed by further washing in boiling DI water (10-minute sonication) and finally 5-10 minutes sonication in isopropyl alcohol**.** The substrates were dried with a nitrogen gun and placed in a UV-Ozone cleaner for 10 minutes to ensure removal of any surface particulates.

6.2 Deposition of PEDOT:PSS

PEDOT:PSS solution (Al4083, Ossila) was brought to room temperature, filtered through a 0.45 µm PVDF filter into an amber vial and dynamically spin-coated (45 µL) onto room-temp substrates at 6000 rpm, to achieve a film ~25 nm. The PEDOT:PSS coated substrates were placed on a hotplate at 110 °C for 15 minutes. This was followed by further annealing in the glovebox at 110 °C for 15 minutes to remove any surface moisture. After 15 minutes annealing in the glovebox, the ITO/PEDOT:PSS samples were cooled to room temperature before active layer deposition.

6.3 Active layer deposition

P3HT (Sigma Aldrich) and $PC_{71}BM$ (99% purity, supplied by Ossila) were used as received. P3HT (10mg/mL) was dissolved in chlorobenzene solvent. After heating at 60 °C for 10 minutes followed by cooling towards room temperature, the solution was filtered through a 0.45 µm PTFE filter. The P3HT solution in chlorobenzene was then mixed with 5mg MONs MONs (1:0.5 wt/wt ratio of P3HT:MON) and stirred at 60 °C for an hour. Then 10 mg of $PC_{71}BM$ was added and the final P3HT:MON:$PC_{71}BM$ (1:0.5:1 wt/wt ratio) was heated at 70°C for an hour and cooled to room temperature prior to spin coating. The solutions were then spin-cast onto ITO/PEDOT:PSS at 1000 rpm under a nitrogen atmosphere in a glove box, forming films of ~150 nm as determined by Dektak®. For control devices, 1:1(wt/wt) (P3HT:$PC_{71}BM$) were spin cast at 2000 rpm in the same method as above to give films of ≈150nm. Solvent-vapour annealing of the films was carried out by placing the coated substrates in a sealed metal container containing 20µL of solvent chlorobenzene) for 5 minutes.

6.4 Top contact deposition and encapsulation

The ITO/PEDOT:PSS/active layer substrates were placed under a vacuum of <2×10^{-6} mbar before thermally evaporating BCP (5nm) and Silver (100nm) layers using a shadow mask. The devices were encapsulated using an epoxy resin (Ossila, E131).

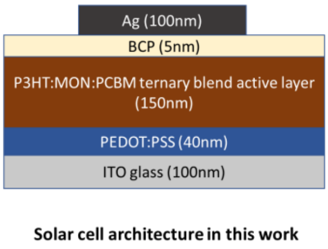

Figure S22. Schematic representation of the device architecture used in this work.

7 Device testing

Device performance was determined under ambient conditions by measuring *J–V* curves using a Newport 92251A-1000 solar simulator, with devices illuminated through a 0.0256 cm^2 aperture mask. Before each set of measurements, the intensity was calibrated to 100 mW cm^{-2} using an NREL certified silicon reference cell. The applied bias was swept from 0.0 to +1.2 V and back again at a scan speed of 0.4 V s^{-1} using a Keithley 237 source measure unit.

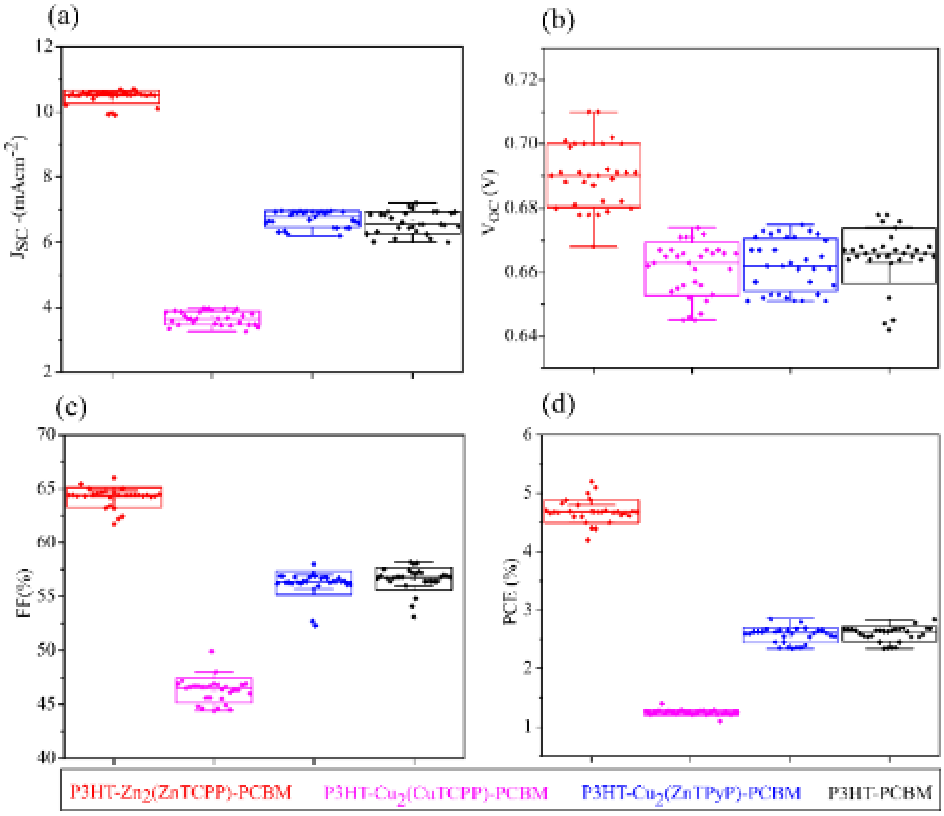

Figure S23. Box plots showing the various device performance parameters for each device configuration (data from 30 devices for each configuration) – see color code at the bottom.

8 Bilayer devices

Bilayer devices were prepared in the same way as the bulk heterojunctions, except for the active layer. Instead of pre-mixing the P3HT-$PC_{71}BM$ and MONs, each component was individually spin-coated orthogonally before the back contact fabrication. This was done to understand the fundamental electronic properties of the MONs that give rise to device function. The devices studied here are inherently limited by the small interfacial area due to the strict bi-layer geometry of the donor/acceptor layer.

8.1 PV parameters of the ITO/PEDOT:PSS/P3HT/Zn_2(ZnTCPP)/BCP/Ag devices

Table S1 PV parameters of the ITO/PEDOT:PSS/P3HT/Zn_2(ZnTCPP)/BCP/Ag devices

Device	Jsc (mA/cm^2)	Voc (V)	FF (%)	PCE (%)
Device#1	-3.52	0.16	26.4	0.15
Device#2	-3.27	0.19	26.68	0.16
Device#3	-3.58	0.16	26.31	0.15
Device#4	-2.92	0.08	25.30	0.06
Device#5	-2.98	0.15	26.13	0.11

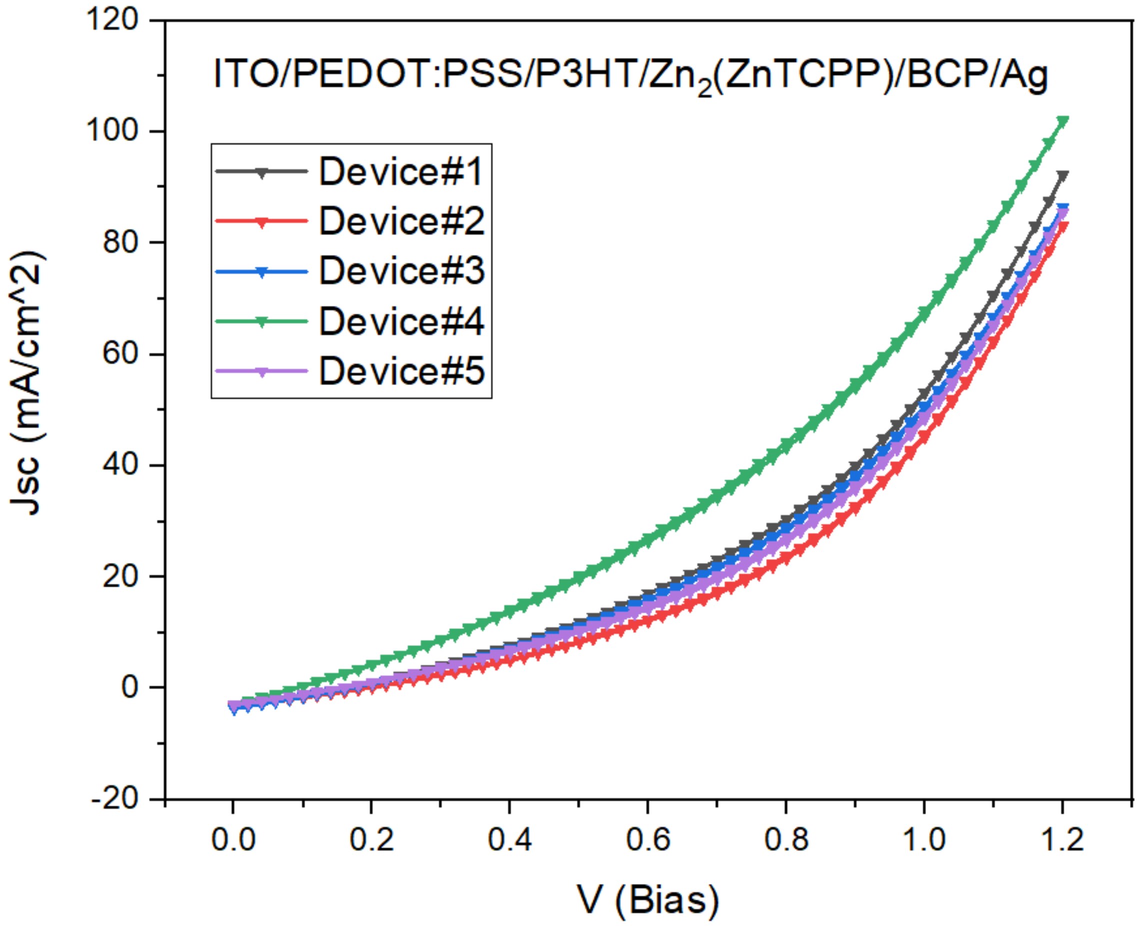

Figure S24. J-V plots of the P3HT-Zn_2(ZnTCPP) bilayer devices.

8.2 PV parameters of the ITO/PEDOT:PSS/Zn_2(ZnTCPP)/$PC_{71}BM$/Ag devices

Table S2 PV parameters of the Zn_2(ZnTCPP)-$PC_{71}BM$ bilayer devices

Device	Jsc (mA/cm^2)	Voc (V)	FF (%)	PCE (%)
Device#1	-3.48	0.55	31.94	0.62
Device#2	-3	0.63	37.40	0.71
Device#3	-3.09	0.34	24.94	0.26
Device#4	-3.29	0.64	33.10	0.69
Device#5	-3.32	0.58	29.95	0.58

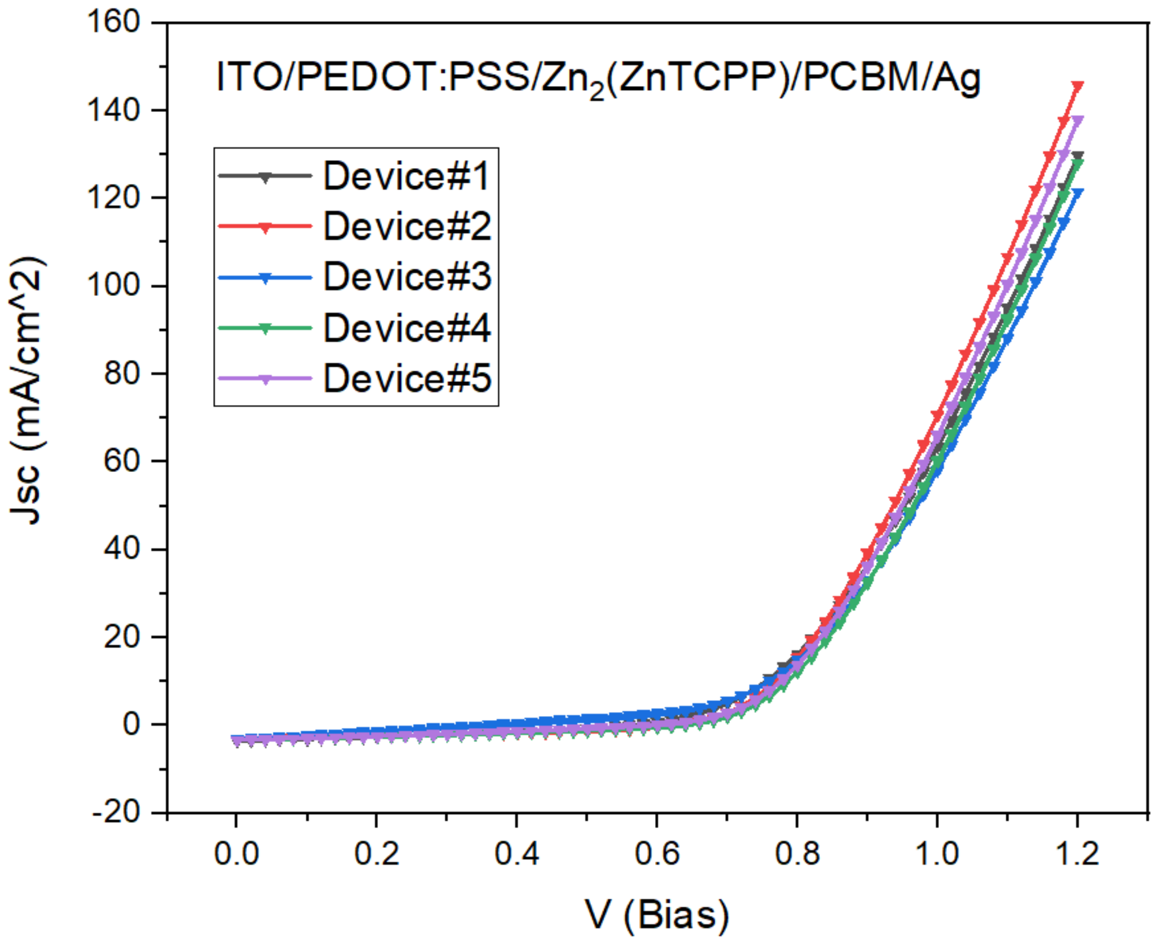

Figure S25. J-V plots of the Zn_2(ZnTCPP)-$PC_{71}BM$ bilayer devices.

8.3 PV parameters of the ITO/PEDOT:PSS/P3HT/Cu_2(CuTCPP)/BCP/Ag devices

Table S3 PV parameters of the P3HT-Cu_2(CuTCPP) bilayer devices

Device	Jsc	Voc	FF	PCE
Device#1	-1.33	0.23	20.72	0.06
Device#2	-1.31	0.24	20.60	0.06
Device#3	-1.42	0.25	20.83	0.07
Device#4	-1.32	0.23	20.80	0.06
Device#5	-1.55	0.23	21.61	0.08

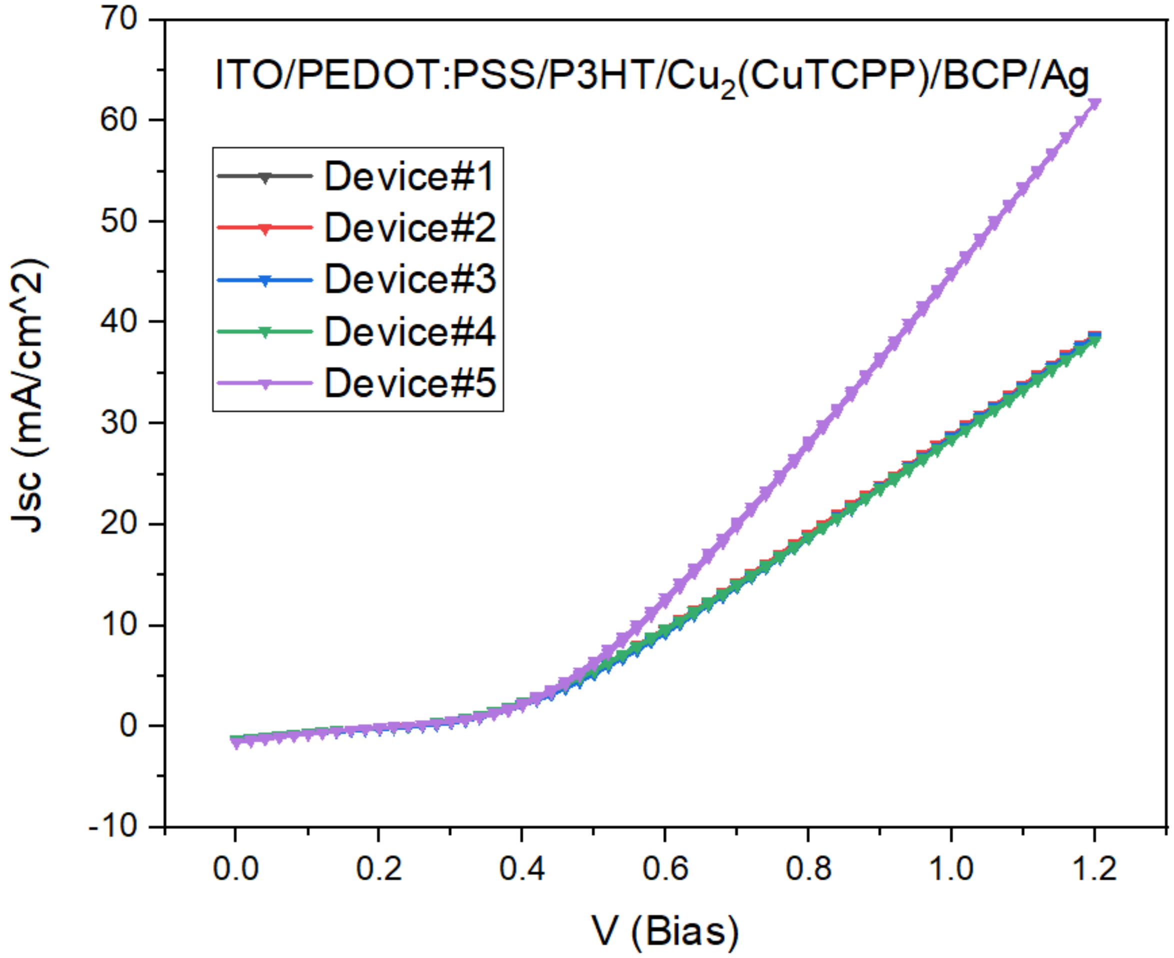

Figure S26. J-V plots of the P3HT-Cu_2(CuTCPP) bilayer devices.

8.4 PV parameters of the ITO/PEDOT:PSS/Cu_2(CuTCPP)/$PC_{71}BM$/Ag devices

Table S4 PV parameters of the Cu_2(CuTCPP)-$PC_{71}BM$ bilayer devices

Device	Jsc	Voc	FF	PCE
Device#1	-1.68	0.04	24.02	0.02
Device#2	-1.81	0.09	24.56	0.03
Device#3	-1.71	0.05	24.56	0.02
Device#4	-1.89	0.05	24.70	0.02
Device#5	-1.35	0.06	24.88	0.02

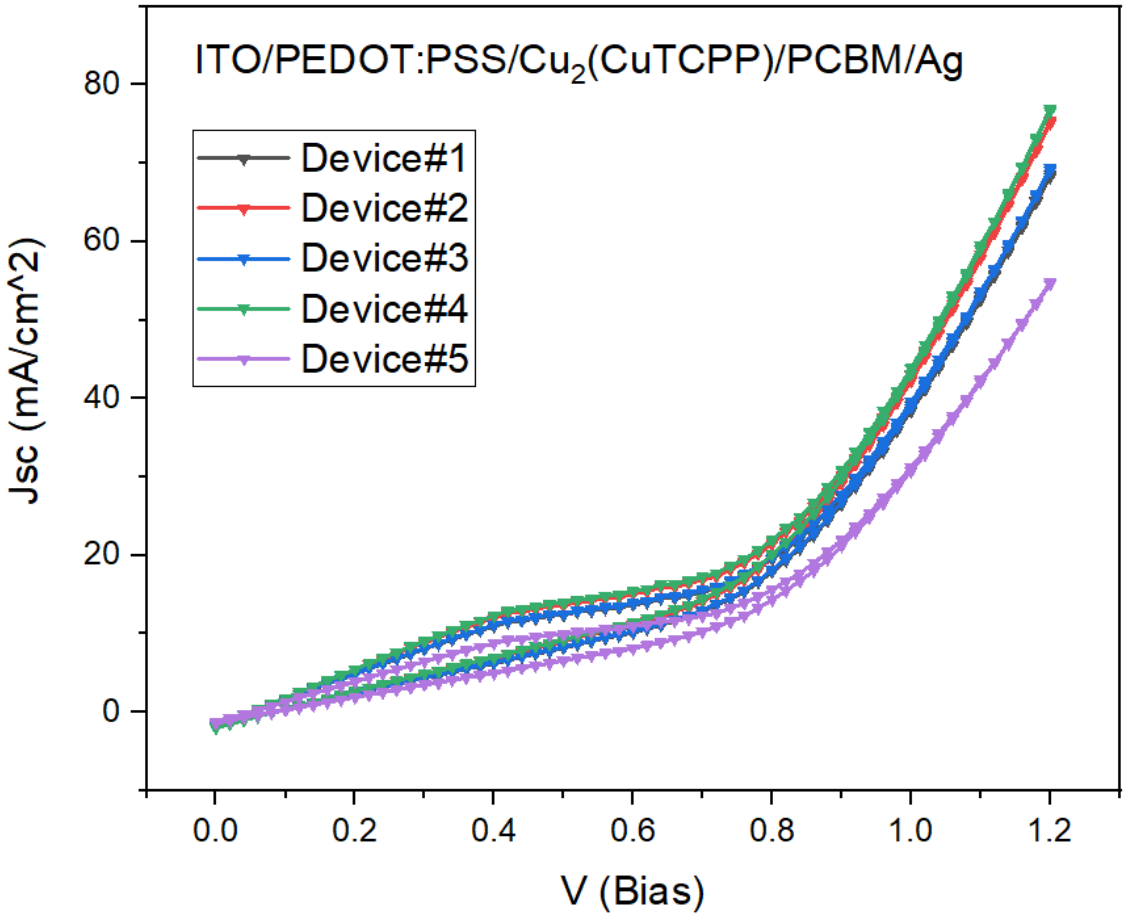

Figure S27. J-V plots of the Cu_2(CuTCPP)-$PC_{71}BM$ bilayer devices.

8.5 PV parameters of the ITO/PEDOT:PSS/P3HT/Cu_2ZnTPyP/BCP/Ag devices

Table S5 PV parameters of the P3HT-Cu_2ZnTPyP bilayer devices

Device	Jsc	Voc	FF	PCE
Device#1	-1.44	0.37	29.51	0.23
Device#2	-1.41	0.31	28.81	0.20
Device#3	-1.42	0.40	29.87	0.18
Device#4	-1.41	0.21	26.57	0.11
Device#5	-1.44	0.43	29.95	0.15

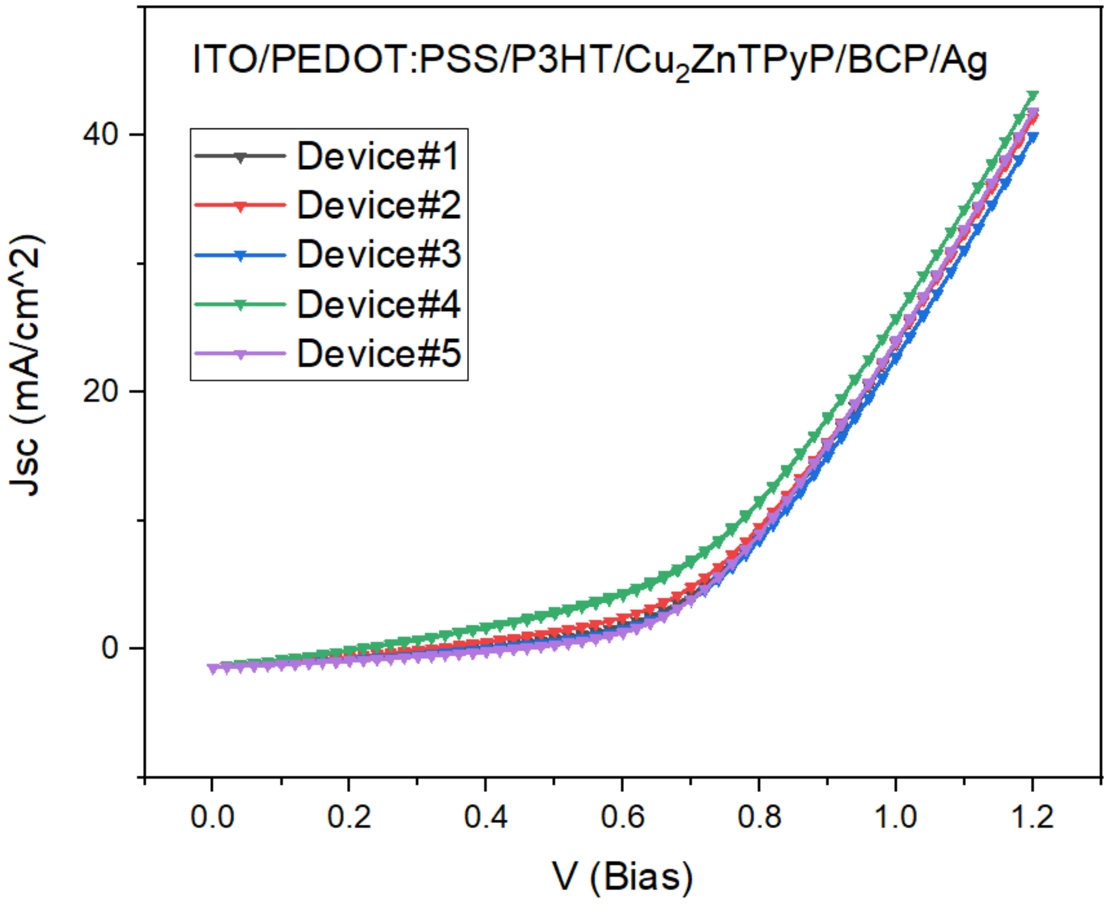

Figure S28. J-V plots of the P3HT-Cu_2ZnTPyP bilayer devices.

8.6 PV parameters of the ITO/PEDOT:PSS/$Cu_2ZnTPyP$/$PC_{71}BM$/Ag devices

Table S6 PV parameters of the $Cu_2ZnTPyP$-$PC_{71}BM$ bilayer devices

Device	Jsc	Voc	FF	PCE
Device#1	-1.88	0.34	20.59	0.13
Device#2	-1.78	0.32	19.86	0.11
Device#3	-1.75	0.32	19.80	0.11
Device#4	-1.74	0.19	24.00	0.07
Device#5	-1.75	0.32	20.44	0.11

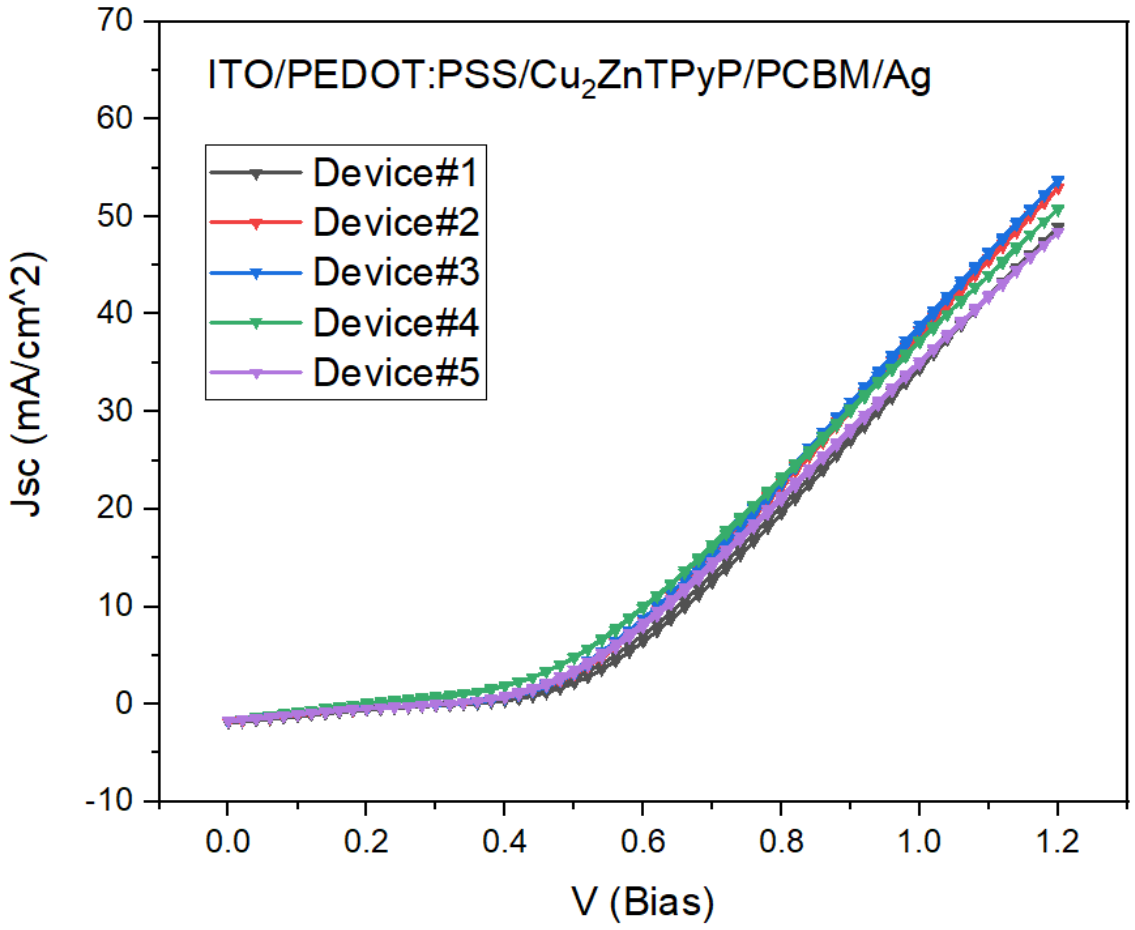

Figure S29. J-V curves of the $Cu_2ZnTPyP$-$PC_{71}BM$ bilayer devices

9 Photoelectron spectroscopy investigations

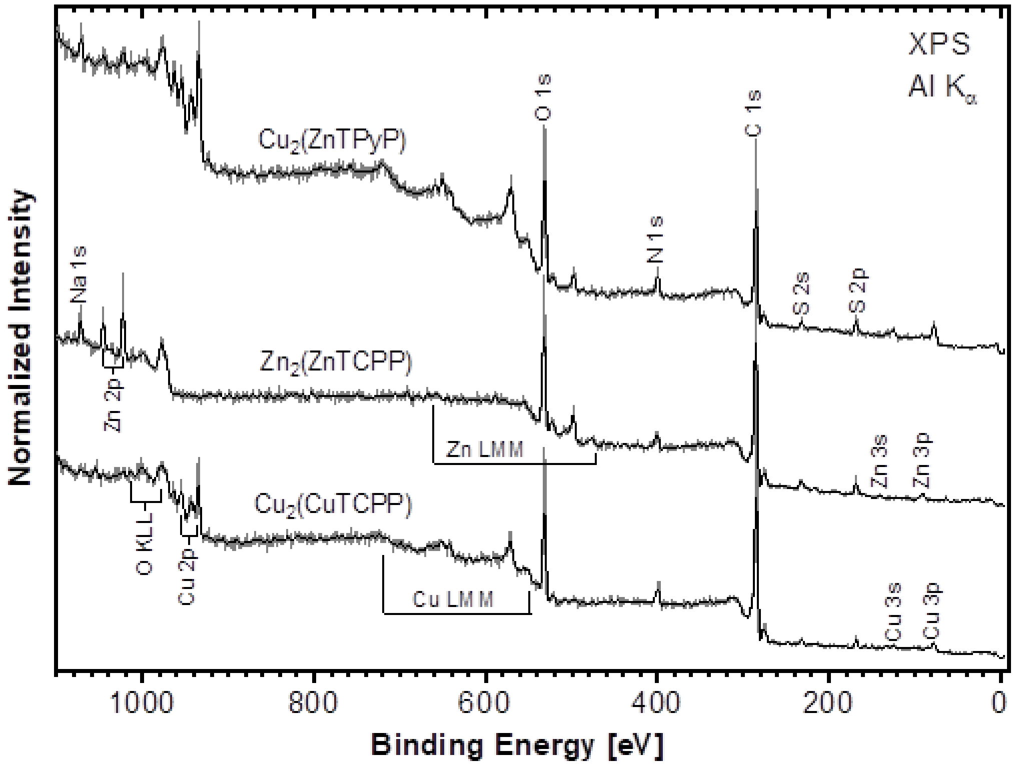

Figure S30. XPS survey spectra of different MONs/PEDOT:PSS samples. Normalized raw data is shown in grey and slightly smoothed (5 point FFT filter) data is shown as black solid lines. The most prominent photoemission and Auger lines are labelled. Offsets were added for clarity.

The XPS survey spectra of the MONs/PEDOT:PSS samples (see **Figure S30**) exhibit all MONs related photoemission (and Auger) lines. In addition, PEDOT:PSS related signals can be observed (most prominently the S 2p signal at 161 eV BE), indicating an incomplete coverage of the substrate. Based on the lower intensity of the XPS features related to S in the XPS survey spectrum of the Cu_2(CuTCPP), it can be concluded that the MONs coverage of the PEDOT:PSS, with a layer exceeding in thickness the XPS information depth (in this case the S 2p photelectron inelastic mean free path is approx. 3 nm [QUASES-IMFP-TPP2M Ver. 3.0 Inelastic electron mean free paths calculated from the TPP-2M formula: S. Tanuma, C. J. Powell, D. R. Penn: Surf. Interf. Anal. 21 (1994) 165 & H. Shinotsuka, S. Tanuma, C. J. Powell, D. R. Penn:

Surf. Interf. Anal. 47 (2015) 871]), is best for Cu_2(CuTCPP) – in agreement with the topography analysis in conjunction with Figure 1 in the manuscript.

The Na 1s line that can be observed at 1071 eV BE (in particular for the Zn_2(ZnTCPP) and Cu_2(ZnTPyP) samples) can be explained by out diffusion of Na from the glass substrate promoted by the thermal treatment of the PEDOT:PSS.

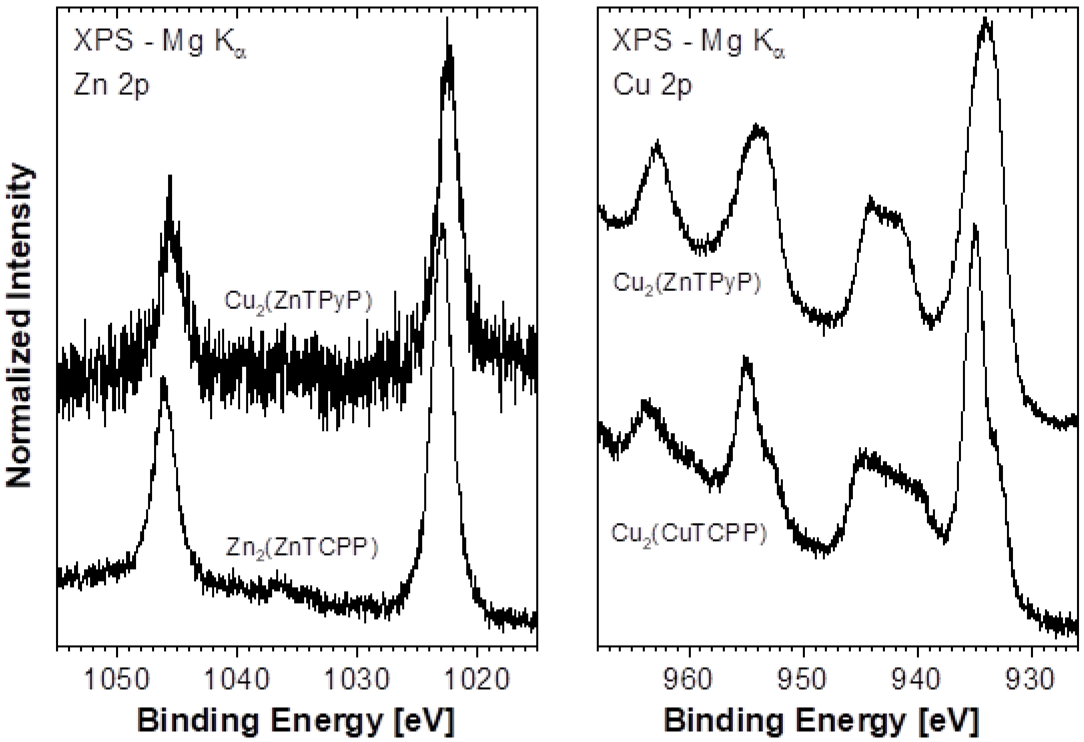

Figure S31. Zn 2p (left panel) and Cu 2p (right panel) XPS detail spectra of different MONs/PEDOT:PSS samples. Offset was added for clarity. The significantly lower signal-to-noise level of the Cu_2(ZnTPyP) MONs Zn 2p spectrum is due to the much lower amount of Zn in this sample compared to the Zn_2(ZnTCPP) MONs.

The Zn 2p and Cu 2p detail spectra in **Figure S31** clearly indicate the deposition of the Zn_2(ZnTCPP) and Cu_2(ZnTPyP) as well as of the Cu_2(CuTCPP) and Cu_2(ZnTPyP) MONs on the PEDOT:PSS, respectively. Close inspection of the spectra reveals that the Zn 2p spectrum of the Cu_2(ZnTPyP) MONs is shifted to lower BE by approx. 0.5 eV compared to that of the Zn_2(ZnTCPP) MONs sample. We find a similar spectral shift when comparing the Cu 2p spectra of the Cu_2(ZnTPyP) and Cu_2(CuTCPP) MONs. In addition, in this case also the spectral shape of the photoemission line is significantly different. While the Cu 2p spectrum of the Cu_2(ZnTPyP)

agrees very well with the spectral fingerprint of Cu in a +2 oxidation state ,[1] the spectrum of Cu_2(CuTCPP) MONs seems rather to be a mixture of Cu in oxidation states of +1 and +2 ,[1] in accordance with previous reports.[2-3]

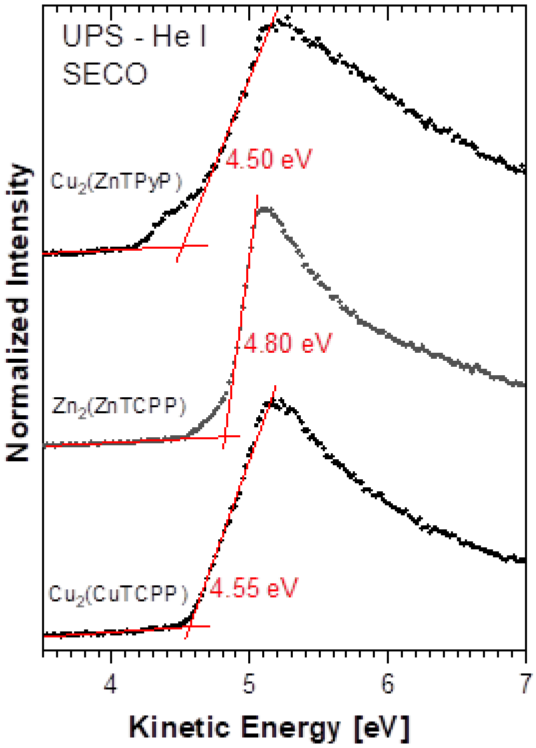

Figure S32a. UPS detail spectra of the secondary electron cut-off (SECO) region for the studied different MONs/PEDOT:PSS samples together with the linear extrapolations of the leading edges to derive the corresponding work function (Φ) values (also indicated). The experimental uncertainty of the Φ determination was estimated to be ±0.05 eV. Offsets were added for clarity.

Since the VBM values for Cu_2(CuTCPP), Zn_2(ZnTCPP), and Cu_2(ZnTPyP) are very similar (see Figure 4b), also the energetic landscape within the ternary blend active layer is presumably very similar for the three different MONs. This is supported by the derived ionization energies (IE). **Figure S32a** shows the UPS spectra of the SECO region for the studied different MONs/PEDOT:PSS samples. In the SECO spectra of Zn_2(ZnTCPP) and Cu_2(ZnTPyP) significant spectral intensity below the main onset can be observed, which we ascribe to topography (see Figure 1 and related discussion in the manuscript) and the inability of the MONs to completely cover the PEDOT:PSS substrate (see XPS survey spectra above),

presumably affecting the samples SECO spectra by differential charging effects. Linear extrapolation of the leading (main) edges results in work function (Φ) values of 4.55, 4.80, and 4.50 (±0.05) eV for the for Cu_2(CuTCPP), Zn_2(ZnTCPP), and Cu_2(ZnTPyP) MONs, respectively. Together with the derived VBM values of 0.3, 0.4, and 0.4 (±0.1) eV (indicated by the red arrows in Figure 4b), we compute the ionization energies to be 4.85, 5.2, and 4.9 (±0.11) eV for Cu_2(CuTCPP), Zn_2(ZnTCPP), and Cu_2(ZnTPyP), respectively, again indicating a very similar electronic structure of the different MONs. Close inspection of the IE values reveals that the ones for Cu_2(CuTCPP) and Cu_2(ZnTPyP) actually agree (within the experimental uncertainty) while the IE of Zn_2(ZnTCPP) MONs seems to be slightly (but significantly) larger. Taking the electronic structure of P3HT and $PC_{71}BM$ into account,[4] all MONs considered here are suitable as ternary additives in a P3HT:$PC_{71}BM$ blend as the resulting energy level alignment in the ternary blend is presumably beneficial for hole transport. The optical band gaps of the three MONs are shown below in **Figure S32b**.

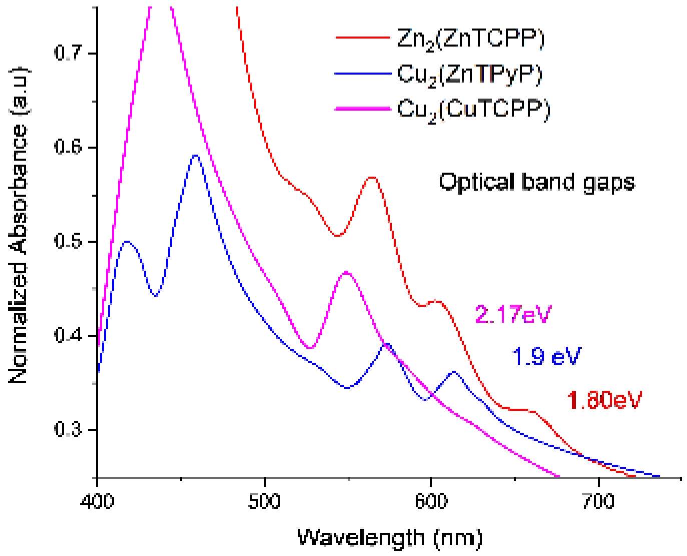

Figure S32b. Optical band gaps of the three MONs used in this study.

10 AFM imaging of ternary bulk heterojunctions and Fast Fourier Transform (FFT) analysis for grain size calculation

10.1 P3HT-$PC_{71}BM$

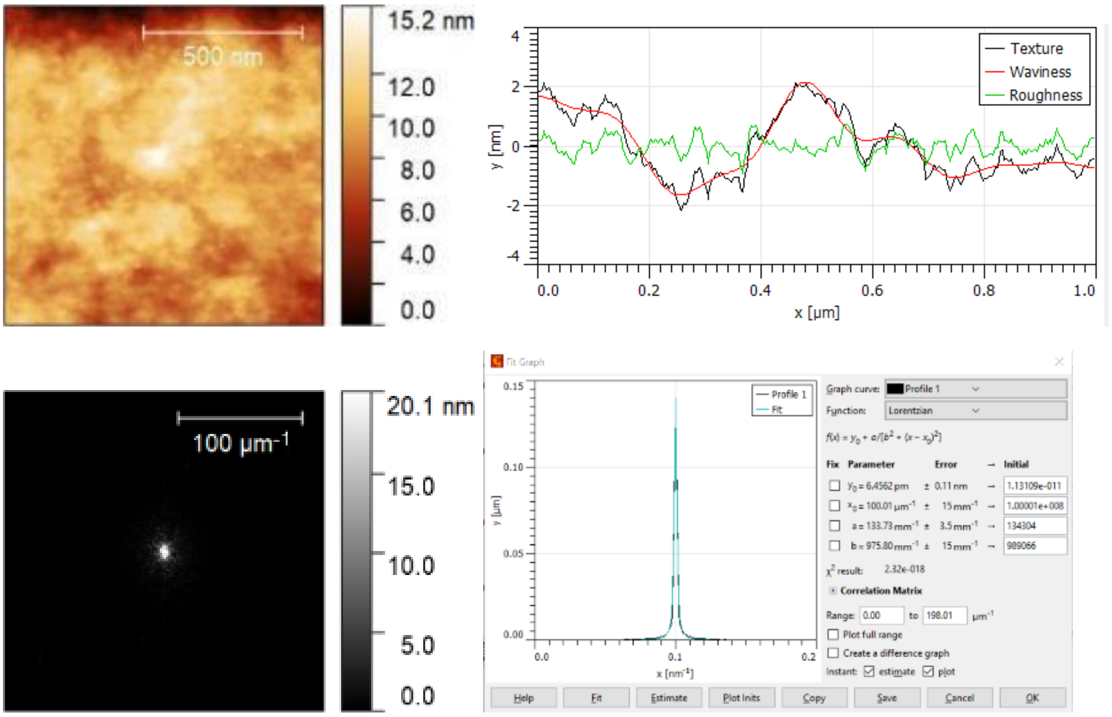

Figure S33. AFM and FFT analysis on P3HT-$PC_{71}BM$ reference sample.

10.2 P3HT-Zn_2(ZnTCPP)-$PC_{71}BM$

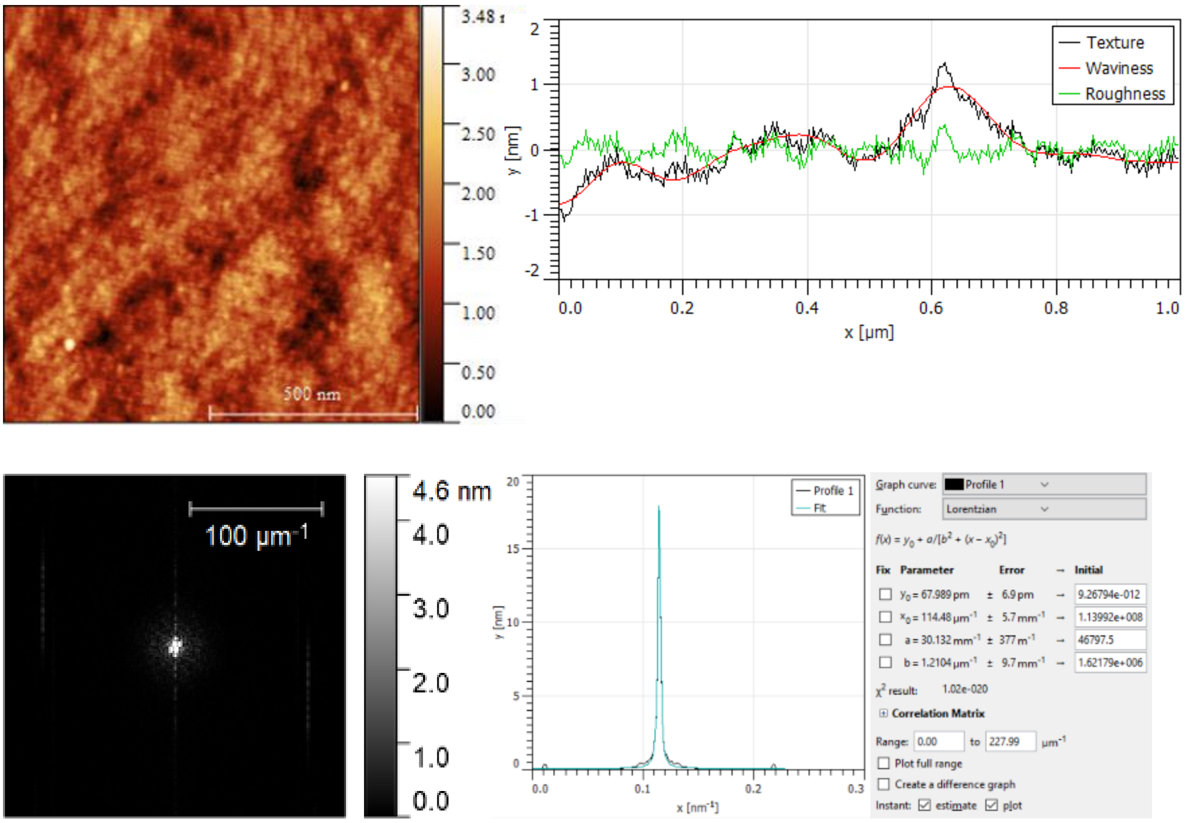

Figure S34. AFM and FFT analysis on P3HT-Zn_2(ZnTCPP)-$PC_{71}BM$ reference sample.

10.3 P3HT-Cu_2(CuTCPP)-$PC_{71}BM$

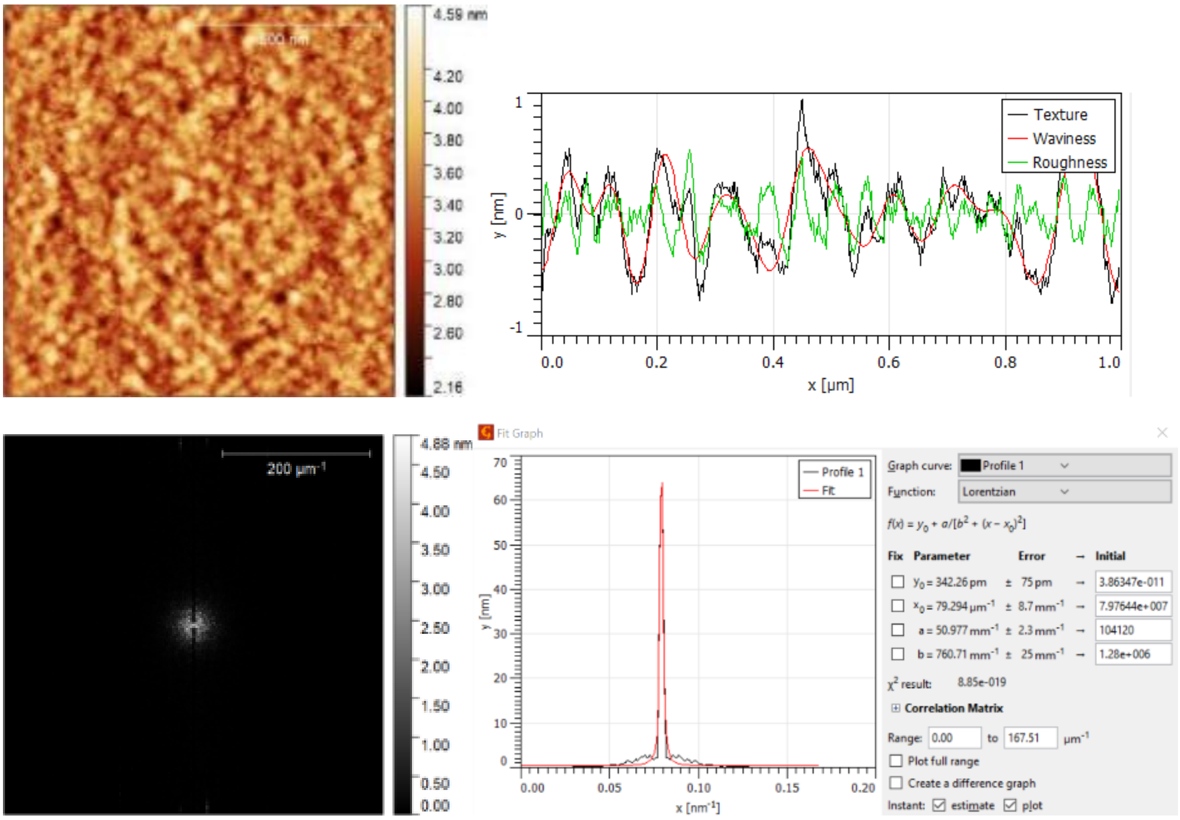

Figure S35. AFM and FFT analysis on P3HT-Cu_2(CuTCPP)-$PC_{71}BM$ reference sample.

10.4 P3HT-Cu_2(ZnTPyP)-PC_{71}BM

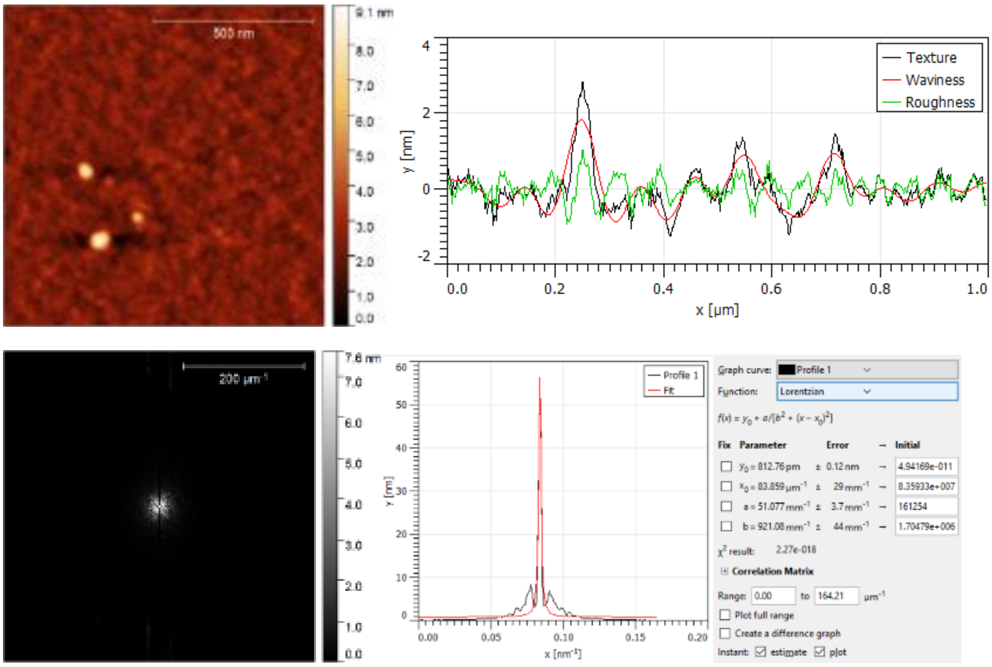

Figure S36. AFM and FFT analysis on P3HT-Cu_2(ZnTPyP)-PC_{71}BM reference sample.

Table S7 Summary of derived grain size values

Device active layer	FFT – Inverse space value	Calculated grain size
P3HT-$PC_{71}BM$	100 μm^{-1}	10 nm
P3HT-Zn_2(ZnTCPP)-$PC_{71}BM$	115 μm^{-1}	8.7 nm
P3HT-Cu_2(CuTCPP)-$PC_{71}BM$	21.09 μm^{-1}	47.4 nm
P3HT-Cu_2(ZnTPyP)-$PC_{71}BM$	48.9 μm^{-1}	20.4 nm

11 Raman microspectroscopy

11.1 Microstructure mapping

The Raman mapping of the film microstructure corresponding to the 1447 cm^{-1} symmetric C=C stretch is shown in the figure S37. The yellow domains represent the highest intensity and therefore the highest relative P3HT concentration, and the black domain represent the lowest P3HT concentration.

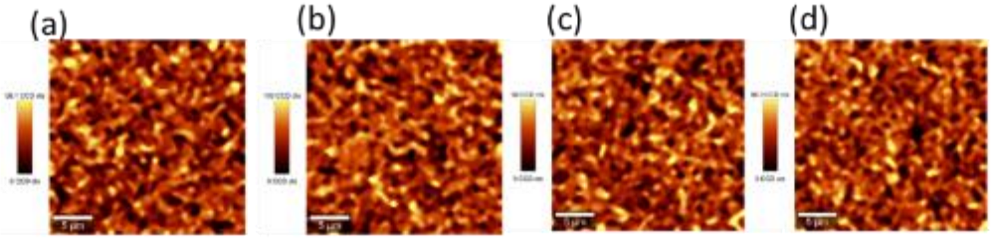

Figure S37. Raman microstructure of the device films – (a) P3HT-$PC_{71}BM$ reference; (b) P3HT-Zn_2(ZnTCPP)-$PC_{71}BM$, (c) P3HT-Cu_2(CuTCPP)-$PC_{71}BM$, (d) P3HT-Cu_2(ZnTPyP)-$PC_{71}BM$.

11.2 True component analysis

Multivariate analysis based on automatic component identification - This method is based on the true component analysis function of the WiTec program where the software was asked to select a first spectrum from the whole dataset (the most intense – in this case P3HT) as a base spectrum and calculate the residual image.[5] If the residual image is structured, subsequent cycles of adding components automatically and calculating the corresponding residual images are performed until it was considered that the residual image is only noise. The components automatically detected were then demixed, compared with the reference spectra. The obtained colour maps are shown below where the MON regions are represented in red, while the P3HT-PCBM regions are coloured in blue and green respectively.

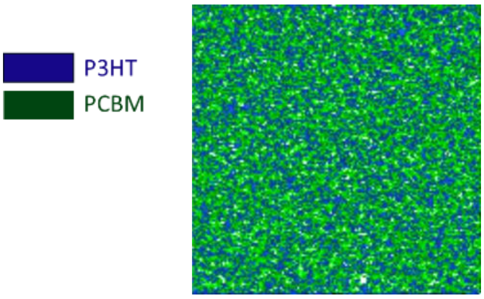

Figure S38. Component colour maps after true component analysis for the P3HT-$PC_{71}BM$ blend.

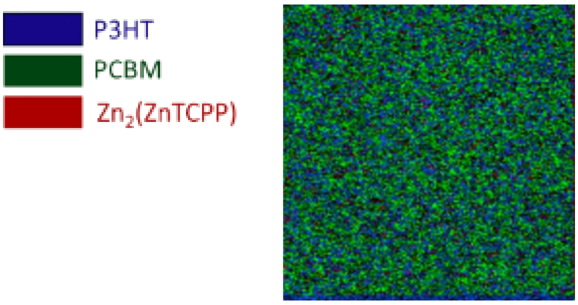

Figure S39. Component colour maps after true component analysis for the P3HT-Zn_2(ZnTCPP)-PC_{71}BM blend.

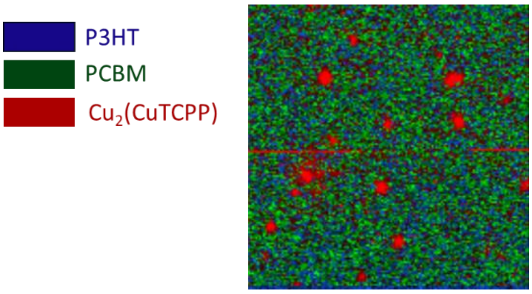

Figure S40. Component colour maps after true component analysis for the P3HT-Cu_2(CuTCPP-)PC_{71}BM blend.

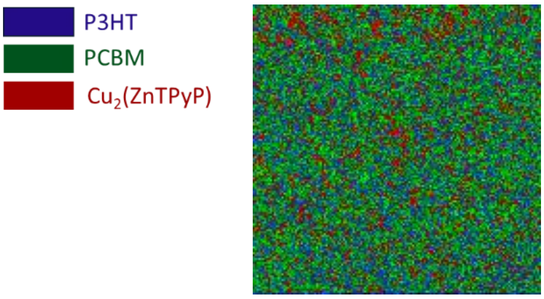

Figure S41. Component colour maps after true component analysis for the P3HT-Cu_2(ZnTPyP)-PC_{71}BM blend.

12 Device stability

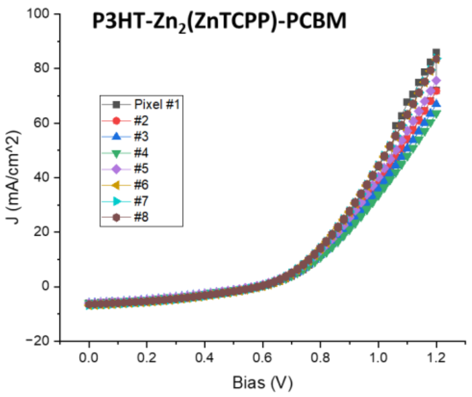

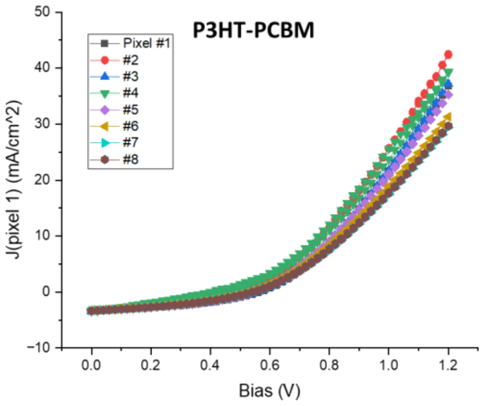

Figure S42. J-V Curves of P3HT-Zn2(ZnTCPP)-$PC_{71}BM$ and P3HT-$PC_{71}BM$ stored in air, room temperature for a month.

Table S8 – Device performance data for P3HT-Zn_2(ZnTCPP)-$PC_{71}BM$ device after 1 month outside glovebox

Parameter	Pixel 1	Pixel 2	Pixel 3	Pixel 4	Pixel 5	Pixel 6	Pixel 7	Pixel 8
Voc (V)	0.595229	0.595091	0.580696	0.579579	0.568364	0.593788	0.592932	0.589474
Jsc (mA/cn	-6.06641	-6.16406	-6.00391	-5.93359	-6.10938	-6.82422	-6.80078	-6.51172
FF (%)	35.1994	34.7946	33.5586	32.5677	33.5868	35.4635	35.3077	35.8804
Vmpp (V)	0.34	0.34	0.32	0.32	0.32	0.34	0.34	0.34
Jmpp (mA/	-3.73828	-3.75391	-3.65625	-3.5	-3.64453	-4.22656	-4.1875	-4.05078
Efficiency	1.27102	1.27633	1.17	1.12	1.16625	1.43703	1.42375	1.37727

Table S9 – Device performance data for P3HT-$PC_{71}BM$ device after 1 month outside glovebox

Parameter	Pixel 1	Pixel 2	Pixel 3	Pixel 4	Pixel 5	Pixel 6	Pixel 7	Pixel 8
Voc (V)	0.56625	0.47619	0.558519	0.397358	0.547742	0.527848	0.544938	0.545366
Jsc (mA/cn	-3.39453	-3.4375	-3.40234	-3.39844	-3.34375	-3.25	-3.31641	-3.35156
FF (%)	39.287	32.1395	37.6714	27.6192	39.0857	36.7239	36.8916	37.13
Vmpp (V)	0.36	0.26	0.34	0.22	0.34	0.32	0.34	0.34
Jmpp (mA/	-2.09766	-2.02344	-2.10547	-1.69531	-2.10547	-1.96875	-1.96094	-1.99609
Efficiency	0.755156	0.526094	0.715859	0.372969	0.715859	0.63	0.666719	0.678672

13 References

1 J. F. Moulder, W. F. Stickle, W. M. Sobol and K. D. Bomben, 1992.

2 S. Zhao, S. Li, Z. Zhao, Y. Su, Y. Long, Z. Zheng, D. Cui, Y. Liu, C. Wang, X. Zhang and Z. Zhang, *Environ. Sci. Pollut. Res.*, , DOI:10.1007/s11356-020-09865-z.

3 S. Qiu, Y. Su, H. Zhao, L. Wang and Q. Xu, *Corros. Sci.*, 2021, **178**, 109090.

4 N. E. Widjonarko, E. L. Ratcliff, C. L. Perkins, A. K. Sigdel, A. Zakutayev, P. F. Ndione, D. T. Gillaspie, D. S. Ginley, D. C. Olson and J. J. Berry, *Thin Solid Films*, 2012, **520**, 3813–3818.

5 I. Benito-González, M. Martínez-Sanz, A. López-Rubio and L. G. Gómez-Mascaraque, *J. Raman Spectrosc.*, 2020, **51**, 2022–2035.
